# Estimating the health burden of road traffic injuries in Malawi using an individual-based model

**DOI:** 10.1186/s40621-022-00386-6

**Published:** 2022-07-12

**Authors:** Robert Manning Smith, Valentina Cambiano, Tim Colbourn, Joseph H. Collins, Matthew Graham, Britta Jewell, Ines Li Lin, Tara D. Mangal, Gerald Manthalu, Joseph Mfutso-Bengo, Emmanuel Mnjowe, Sakshi Mohan, Wingston Ng’ambi, Andrew N. Phillips, Paul Revill, Bingling She, Mads Sundet, Asif Tamuri, Pakwanja D. Twea, Timothy B. Hallet

**Affiliations:** 1 University College London, Gower Street, London, WC1E 6BT UK; 2 Imperial College London, South Kensington Campus, London, SW7 2AZ UK; 3 Ministry of Health and Population, P.O. Box 30377, Lilongwe 3, Malawi; 4 Kamuzu University of Health Sciences, Mahatma Gandhi, 52X8+782 Blantyre, Malawi; 5 University of Malawi, J86P+8V Zomba, Malawi; 6 University of York, York, YO10 5DD UK; 7 REMEDY-Center for treatment of Rheumatic and Musculoskeletal Diseases, Diakonhjemmet Hospital, Oslo, Norway

**Keywords:** Road traffic injuries, Malawi, Individual-based model, Health burden

## Abstract

**Background:**

Road traffic injuries are a significant cause of death and disability globally. However, in some countries the exact health burden caused by road traffic injuries is unknown. In Malawi, there is no central reporting mechanism for road traffic injuries and so the exact extent of the health burden caused by road traffic injuries is hard to determine. A limited number of models predict the incidence of mortality due to road traffic injury in Malawi. These estimates vary greatly, owing to differences in assumptions, and so the health burden caused on the population by road traffic injuries remains unclear.

**Methods:**

We use an individual-based model and combine an epidemiological model of road traffic injuries with a health seeking behaviour and health system model. We provide a detailed representation of road traffic injuries in Malawi, from the onset of the injury through to the final health outcome. We also investigate the effects of an assumption made by other models that multiple injuries do not contribute to health burden caused by road accidents.

**Results:**

Our model estimates an overall average incidence of mortality between 23.5 and 29.8 per 100,000 person years due to road traffic injuries and an average of 180,000 to 225,000 disability-adjusted life years (DALYs) per year between 2010 and 2020 in an estimated average population size of 1,364,000 over the 10-year period. Our estimated incidence of mortality falls within the range of other estimates currently available for Malawi, whereas our estimated number of DALYs is greater than the only other estimate available for Malawi, the GBD estimate predicting and average of 126,200 DALYs per year over the same time period. Our estimates, which account for multiple injuries, predict a 22–58% increase in overall health burden compared to the model ran as a single injury model.

**Conclusions:**

Road traffic injuries are difficult to model with conventional modelling methods, owing to the numerous types of injuries that occur. Using an individual-based model framework, we can provide a detailed representation of road traffic injuries. Our results indicate a higher health burden caused by road traffic injuries than previously estimated.

## Background

Road traffic injuries (RTIs) are a significant contributor to death and disability globally (Vos et al. 2015; Lozano et al. 2012). The health burden caused by RTIs is increasingly significant, with RTIs being ranked within the top ten causes of disability adjusted life years (DALYs) (Bhalla et al. 2013), with the Global Burden of Disease (GBD) study estimating RTIs caused 73 million DALYs globally in 2019 (Abbafati et al. 2020). The World Health Organization (WHO) estimates that 93% of the global fatalities due to RTIs occur in low and middle-income countries (LMICs), despite these regions only accounting for 60% of vehicles worldwide (WHO 2015). Despite the increasing significance of RTIs in human health, RTIs receive significantly less research attention than other health conditions (Lagarde 2007).

In Malawi, the incidence of injury and mortality due to road traffic crashes is estimated to have increased over time from 135,000 RTIs in 2010 to 180,000 in 2019 (Global Health Data 2017), likely due to increased vehicle ownership (Banza et al. 2018). Consequently, the health burden caused by RTIs is expected to increase (Mathers and Loncar 2006). The majority of road injuries occur in young adult males in Malawi (Banza et al. 2018; Schlottmann et al. 2017). RTIs are a preventable health burden with well-established preventative measures such as improvement to road infrastructure, robust driver licencing or enforcement of traffic laws. Some preventative measures are present in Malawi’s road safety laws (Government of Malawi 1997); however, there is reportedly limited compliance (Ngwira et al. 2020; Sundet et al. 2020).

It is difficult to determine the extent of health burden and the burden on the health system imposed by RTIs in Malawi. The most recent data-based estimate for the incidence of RTI in Malawi comes from the 2003 World Health Survey (WHO 2005), estimating 3562 RTIs per 100,000 person years (see ‘Appendix’ for calculation). The only other available estimate measured per 100,000 person years for the incidence of RTIs comes from the GBD study, who used the results of the 2003 World Health Survey in Malawi to inform their estimated incidence of RTIs, estimating the incidence of RTIs which warrant some form of medical intervention rather than all injuries. There is limited reporting on the use of the health system by RTI patients in Malawi outside of referral hospitals, but a recent study pooling the available data from several trauma registries in Malawi has shown that the majority (48%) of trauma-related admissions are for RTI patients (Chokotho et al. 2022).

The most commonly available metrics regarding RTIs concern only the incidence of mortality, and these are often not consistent between different sources. There is no centralised trauma care or reporting systems in place in Malawi, meaning that there is limited information on the national provision of care and epidemiology of injuries in Malawi (Samuel et al. 2012). Data from Malawi’s government run integrated household surveys (IHS) can be used to formulate an estimate for the incidence of mortality due to road injuries in Malawi. The most recent survey estimates 16.8 deaths per 100,000 person years occur due to RTIs (National Statistical Office 2021) (see ‘Appendix’ for calculation). Police and hospital records of road traffic injury fatalities exist for Malawi, with hospital and police records estimating 5.1 and 7.5 deaths per 100,000 person years, respectively. However, these records are incomplete (Samuel et al. 2012). A capture–recapture analysis aiming to account for the likelihood of underreported RTIs in Malawi estimated an incidence of 19.2–20.9 deaths per 100,000 person years (Samuel et al. 2012). The largest published estimate for the incidence of mortality comes from the WHO, with estimated 35 deaths per 100,000 person years in Malawi (WHO 2015). This lack of clarity regarding the level of mortality makes determining the full extent of the health burden caused by RTIs in Malawi difficult.

Mathematical models of RTIs in Malawi are limited in number. Currently, the most descriptive available model is the GBD study, which provides estimates for the occurrence, demographics and health outcomes (death and DALYs) for those injured in road traffic collisions (Global Health Data 2017). This model makes an important simplifying assumption with regard to injuries, in that multiple injuries aren’t accounted for when predicting the incidence and health consequences of RTIs, and instead the most serious injury was used to represent the majority of the health burden (Haagsma et al. 2016). However, arguably multiple injuries are an important aspect of road traffic injury epidemiology, with the number of injuries, type of injuries and even the bodily location of the injury altering the health outcome of RTI patients (Gabbe et al. 2014; Tyson et al. 2015). By not accounting for multiple injuries, it may be that the health consequences of RTI are not represented fully in the GBD estimates, either in the incidence of injuries predicted or the number of deaths predicted in the model. Another relevant model for RTIs in Malawi comes from the WHO, which only estimates the incidence of mortality from RTIs in a regression model, using several covariates, including lifestyle factors, health care availability and road safety laws (WHO 2018). The WHO model does not estimate the occurrence and health consequences of non-fatal RTIs, meaning the model does not capture the full health burden RTIs in Malawi.

In addition, both the GBD and the WHO model, the role of the health system in determining the mortality of people with RTIs is considered; however, this is represented as one of many covariates within a regression model, relating to health care access and quality. This combines people seeking health care and the health system being able to provide care into a single covariate of the model. This approach does not allow for modelling the direct link between physical constraints to providing health care, such as resource availability/staff and also the reasons behind a person seeking or not seeking health care.

Whilst there are some available data on RTIs in Malawi, such as the IHS data in Malawi, this only shows a portion of the burden caused by RTIs, non-fatal injuries aren’t accounted for.

We aim to: (1) provide an estimate of the full health burden of RTIs in Malawi, estimating the effect of the current health system on the number of deaths and DALYs cause by RTIs in Malawi and (2) understand the importance of the assumption that multiple injuries do not contribute to the health burden overall.

We do so by developing a detailed mathematical model of RTIs in Malawi, using an individual-based model (IBM) framework. We model injuries at a diagnosable level (for example, fractured tibia, laceration to the head, etc.) and predict the injured population’s health experience from the initial accident to their interaction with the health system. We explicitly model factors which would influence an individual’s decision to seek health care and once they seek care, model the availability of the care required.

## Methods

### The Thanzi La Onse model: a brief introduction

We developed a model of RTIs using an IBM approach. The road traffic injury model is part of a collection of models, forming the Thanzi La Onse (TLO) multi-disease population health and health system model. Much of the RTIs model presented in this paper makes use of the existing TLO modelling framework, which we provide a brief description of here. A more in-depth description of the TLO modelling framework can be found at https://www.tlomodel.org/.

In the model, the agents are fictional representatives of individuals of the population of Malawi. Each person has a number of attributes assigned to them which represents the demographic, lifestyle and health characteristics of the population of Malawi. For example, each person has an age and sex assigned to them, a smoking and excessive alcohol consumption status and disease infection statuses. A full list of the attribute assigned to each individual related to the road traffic injury model is given in ‘Appendix’, Table 1. The model's population go through life over a predetermined length of simulation time, over the course of which some will develop or be afflicted by diseases/injuries. Some of those with health problems will seek care at one of the model’s representations of the Malawian health system. Here, the health system is represented as a group of resources available for consumption, such as staff time, bed capacity and physical consumables. A detailed description of the model’s health system can be found (see link above). Whether they seek care or not is dependent on lifestyle and demographic characteristics each person possesses and the disease/injury they are affected by, with the health seeking behaviour model being based studies of health seeking behaviour in Malawian children and adults (Ng’ambi et al. 2020; Ng’ambi et al. 2020).

Those who seek health care interact with the model’s representation of Malawi’s health system. The health system model provides health care to the population at differing facility levels, representing the variety of levels of health care such as village level health facilities, district hospitals, regional hospitals and national hospitals. Each facility level has a finite number of resources which can be used to treat patients. For many diseases modelled, the interaction with the health system will in turn affect the health outcomes for the patient, and this health system–epidemiology interaction is captured with the TLO modelling framework.

### Road traffic injuries model

The RTI model essentially asks a series of questions of the model population each month:

1. Who is injured in a road traffic crash this month?
2. Did the injured persons die on the scene of the crash?
3. If they didn’t die on scene, what injuries did each injured person receive?
4. Did they go on to seek health care for their injuries?
5. If they sought health care for their injuries, what do they need from the health system for their treatment?
6. Based on their choice to seek or not seek health care, what health outcomes did each injured person experience (mortality, morbidity or recovery)?

In an attempt to make simplifications to the model which would not affect the overall level of health burden and health system usage, we do not explicitly model individual vehicle collisions. This means that we do not account for properties of the physical world in our model (e.g. speed, road surface, vehicle type, number of vehicles in a crash, the number of people in an individual crash or the relationship between people involved in crashes).

**Fig. 1:**
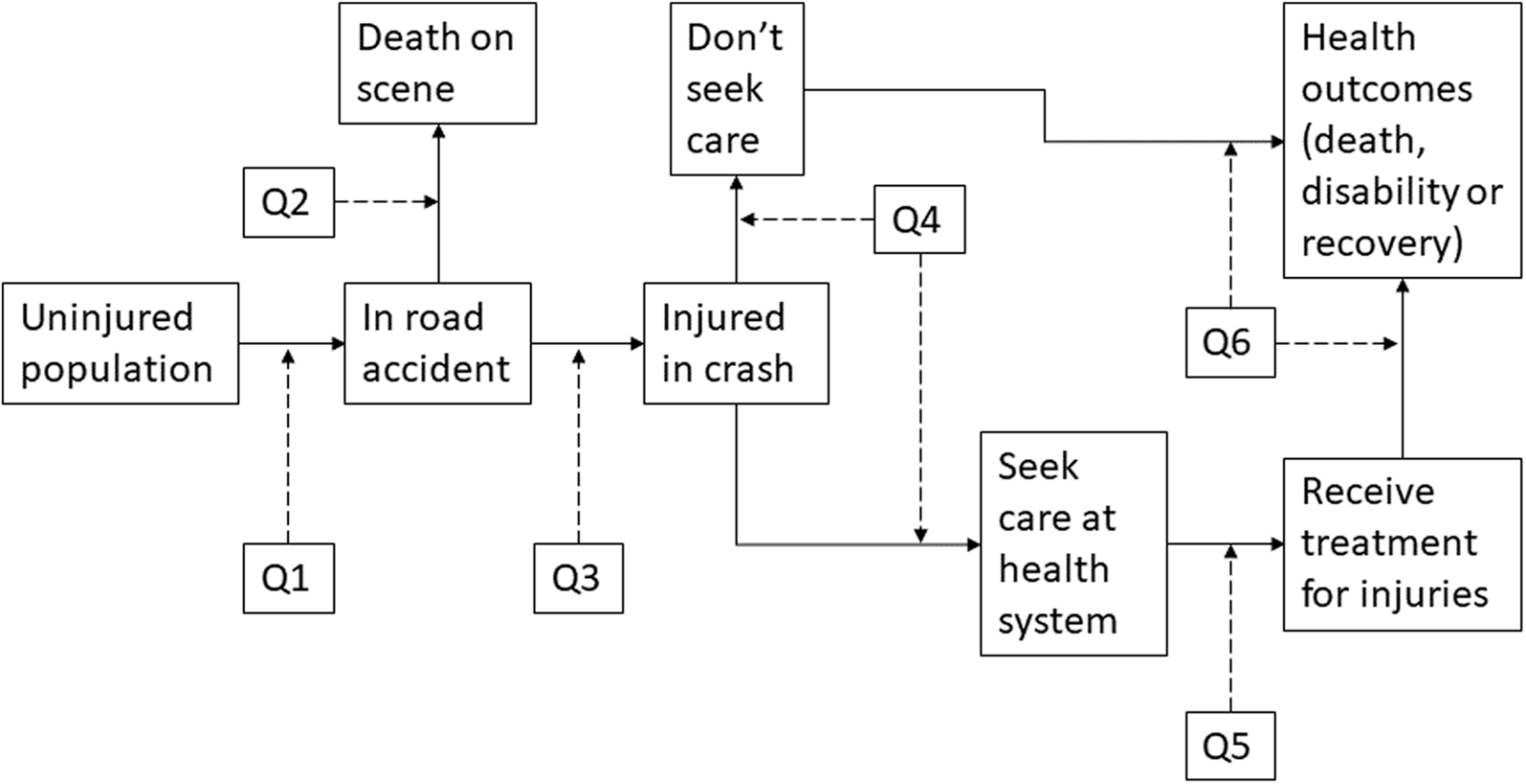
Road traffic injury model diagram, *Q*1 through 6 related to the questions asked by the model to determine the health burden and corresponding health system usage caused by RTIs in Malawi. Owing to the large number parameters used to determine the answer to these questions, we have listed the parameters in ‘Appendix’ in Table 2, organised by the question asked

A schematic of the model is given in Fig. 1, and we go through each question (Q1–Q6) asked by the model in turn below. A full list of parameters is provided in ‘Appendix’. A detailed explanation of the RTI model including the code used in the model can be found on the TLO website.

#### Q1 Who is injured in a road traffic crash this month?

To determine who will be injured in a road traffic collision in the model, we assume that the members of the model’s population are involved in a road traffic collision at fixed rate ‘base_rate_injrti’. We then increase the likelihood of males, those in certain age groups and those who consume excessive alcohol being injured in a road accident. We calibrated the value of the parameter ‘base_rate_injrti’ to produce an incidence of RTIs matching the ten-year average of the GBD’s predicted incidence of RTI. We calibrated the parameter ‘rr_injrti_male’ to the GBD’s estimated gender ratio. The several age-related parameters were also calibrated to the GBD estimated age distribution of those with RTIs. Another risk factor for RTIs is alcohol consumption, for example, roughly 25% of the road traffic injury patients treated at Kamuzu Central Hospital either tested positive for alcohol or reported using alcohol before their injuries (Sundet et al. 2020). We used the relative risk of alcohol consumption in a Tanzanian study to parameterise ‘rr_injrti_excessalcohol’ (Staton et al. 2018).

#### Q2 Did the injured persons die on the scene of the crash?

Some RTIs will invariably be fatal. We assume that pre-hospital mortality occurs in a fixed proportion of those involved in road traffic crashes before the allocation of injuries. The parameter value of ‘imm_death_proportion_rti’ was calibrated to the incidence of on-scene mortality reported by Malawi’s police (Schlottmann et al. 2017).

#### Q3 What injuries did each injured person receive?

In determining the health outcomes for RTIs, the number of injuries, anatomic location of the injury and type of injury have been shown to be important factors in determining mortality and morbidity (Gabbe et al. 2014). The injuries each person receives will also determine what they require from the health system for their treatment. To decide the exact injuries each injured person has, the model assigns injuries in a three-step process. The model determines how many injuries each person has, where the injuries are anatomically located on the body, and based on the anatomic location, what these injuries are. When designing the injury assigning section of the model, we limited the injuries that were assigned to those which would warrant some form of health care. This approach was chosen as some of the injuries received from road accidents will be minor and not produce a significant health burden, an assumption shared with the GBD study (Haagsma et al. 2016).

To determine the number of injuries assigned to each injured person, we developed a negative exponential distribution and calibrated our resulting average number of injuries reported in a paediatric study from Malawi’s Kamuzu Central Hospital (Sundet et al. 2018). Our process of developing the distribution is given in ‘Appendix’. The resulting percentages of single injuries in those with RTIs ranged from 71 to 76%. This falls within the ranges reported in several studies from Sub-Saharan Africa (SSA), with the percentage of single injuries in RTI patients ranging from 66 to 81% (Akinpelu et al. 2007; Ganveer and Tiwari 2005; Madubueze et al. 2010; Sanyang et al. 2017; Thanni and Kehinde 2006).

The anatomic location of the injuries is determined by the average distribution of anatomic injury location found in (Otieno et al. 2004; Ranti et al. 2015), and finally, the exact injury each person receives is informed by several studies. The numerous parameter values used to determine which of the 76 injuries accounted for by the model each person has are given in ‘Appendix’.

#### Q4 Did they go on to seek health care for their injuries?

The model’s predicted health seeking behaviour (HSB) is determined in part by the results of (Ng’ambi et al. 2020; Ng’ambi et al. 2020). The HSB model uses demographic and lifestyle factors, along with the symptoms the person has as a result of their injury/illness to determine HSB. Ng’ambi et al. did not specifically focus on RTIs, instead focusing on injuries in general. We assume that at a certain level of injury, people will always seek health care for their injuries, and we used the injury severity score (ISS) (Baker et al. 1974), to determine this threshold. We assume that there exists a level of injury severity which will cause a person to automatically seek health care, below this level of severity, HSB is determined by the results of Ng’ambi et al. (2020). This severity level is determined by the parameter ‘rt_emergency_care_ISS_score_cut_off’. We established a parameter space which produced an overall level of HSB for RTIs which fell in the bounds reported in other SSA countries (Zafar et al. 2018).

#### Q5 If they sought health care for their injuries, what do they need from the health system for their treatment?

To best represent the provision of treatment for RTIs in Malawi, we based the provided treatments on those that are described in the Malawian treatment guidelines (Ministry of Health 2015), Malawi’s essential health package (EHP) or treatments that have been reported in academic literature. To simplify the model, we assume that if a treatment is described in the treatment guidelines or Malawi-based academic literature, then the treatment is available at local hospitals.

The treatment plan for some injuries must be determined on an individual basis. An example of this is the use of several treatment methods to treat lower extremity fractures in Malawi (Chagomerana et al. 2017). We account for intricate differences in potential treatment plans with fixed probabilities that certain treatment options will be used for each patient. A full list of treatments being provided by the model and references to show evidence they are used in Malawi is given in ‘Appendix’ (Table 3).

#### Q6 Based on their choice to seek or not seek health care, what health outcomes did each injured person experience (mortality, morbidity or recovery)?

We used the severity of a person’s injuries to determine the mortality with and without seeking health care. To quantify the health burden of a person’s injuries, we use a number of commonly used injury severity metrics. To quantify the severity of singular injuries, we used the abbreviated injury score (AIS) (Gennarelli and Wodzin 2006), using the R library ‘InjurySeverityScore’ to convert ICD-9 diagnosis codes to a corresponding AIS score (D. Tian 2019). To quantify the severity of multiple injuries, we used the injury severity score (ISS) (Baker et al. 1974), which makes use of the AIS score of the person’s injuries. We also used the military abbreviated injury score (MAIS) to quantify the severity of injuries and used this to predict the probability of mortality without medical intervention (Champion et al. 2010). The disability burden posed by RTIs was quantified using DALY weights. Each injury has a corresponding DALY weight, sourced from the GBD study (Salomon et al. 2015), see ‘Appendix’. Where the GBD studies DALY weights was too broad or had missing injuries, we used another source (Gabbe et al. 2016).

For those who seek health care and receive treatment (conditional on its availability), we use the ISS score to determine mortality. There was limited information of the probability of death based on the ISS score in Malawi or elsewhere in Africa, as such we used results from a non-African study to establish a relationship between ISS score and mortality. We used the same score boundaries reported in the study (Kuwabara et al. 2010), but scaled the reported probability of mortality in each ISS score boundary so that the overall in-hospital mortality predicted by the model matched the overall in-hospital mortality reported in a national-scale Tanzanian study (Sawe et al. 2021).

For those who did not seek health care and when health care was not available, we assume that mortality is only considered for those with an injury above a certain threshold. In these circumstances, we use their MAIS score to determine mortality. This assumption that the MAIS can be used to predict mortality without health care is tenuous; however, information is limited for the probability of mortality from injuries without medical intervention and this was one of the few mortality-predicting scoring systems in a setting with limited health care provision. The person’s MAIS score corresponded to a probability of mortality, if the person hadn’t sought care after a week of model simulation time, the model used this probability to determine whether they had died from their injuries.

For morbidity, we used DALY weights to quantify health burden from each injury. This health burden was applied to the person when the person’s injuries were assigned. Once a person had sought health care, after a period of recovery time post-treatment this DALY weight was removed (if applicable as some injuries have an associated long-term DALY weight). For those who didn’t seek health care and survived their injuries, we assumed that the DALY weight associated with their injuries would still be removed, but after a longer duration than if they had sought health care for their injuries. The duration of time which a person experiences a health burden associated with their injuries is dependent on the injuries sustained, and a full list of the assumed heal time associated with each injury is given in ‘Appendix’.

A full list of injuries modelled, the health burden associated with the injury, the treatment used, properties of the population modelled and parameters used in the model and their source/calibration process is given in ‘Appendix’.

### Calibration

To calibrate the sections of our model described above, we created multiple scenarios of road traffic injury epidemics. The multiple injury scenario is designed to provide a more realistic representation of Malawi’s RTI epidemic. This scenario is a model based on more realistic assumptions than are currently present in other models of RTIs, such as the GBD model. This scenario is used to produce our estimated incidence of mortality and DALYs.

We want to demonstrate the effect of considering multiple injuries in RTI models, but as our methodology differs greatly from the GBD single injury model, we cannot compare the two models directly to draw conclusions on the effect of modelling multiple injuries. To demonstrate what differences considering multiple injuries in road traffic injuries have on population health, we need to see what our model’s predicted health burden would be without considering multiple injuries. To do this, we created a single injury form of the model, which we use to the estimate resulting incidence of mortality and DALYs and form a point of comparison to the multiple injury model’s results. In both scenarios, we allow the health system to run as normal, representing Malawi’s current care capacity.

We also wish to show the current effectiveness of Malawi’s health system in treating RTIs. We use our most realistic scenario, where we consider multiple injuries from RTIs to represent Malawi’s RTI epidemic with the model’s normal representation of Malawi’s health system. We compare this scenario to one where people experience multiple injuries and do not receive treatment from the health system.

In calibrating each scenario, a particular parameter was incrementally changed, producing a change in the model’s behaviour in an area we desired to calibrate. For example, a change in the parameter ‘rr_injrti_male’ would either increase or decrease the relative risk of being injured in a road collision if male and would in turn change the overall percentage of those in RTIs who are male.

For each scenario and associated parameter value, we ran the model multiple times over ten years of simulation time. The average results of the runs associated with each scenario are an indication of the model’s behaviours for that parameter value. By incrementally changing the parameter values used in the model and taking the average model output per parameter value, we found parameter values which produced model output that matched our various calibration targets, stated in the previous section.

Some parameters were independent of other model parameters, and as such, we could find a single parameter value which produced our calibration target. Other parts of the model were interdependent and required scenarios where multiple parameters were changed in combination with one another to find parameter value which produced calibration targets in each model area.

For some sections of the model, we had no particular estimate to calibrate the model to. For example, we had a range of values for the overall percentage of HSB, rather than a specific percentage of HSB to calibrate to. In this case, we established a parameter space for the parameter ‘rt_emergency_care_ISS_score_cut_off’ which produced an overall level of HSB which fell within our calibration targets.

### Accounting for uncertainty

There is an inherent uncertainty in IBM that is caused by the stochasticity between runs. Each model run is unique, meaning that in order to produce clearer picture of the model’s behaviour, we have to run the model multiple times, taking the average of the results produced in each run. In each scenario, we ran the model with a population of 20,000 for ten years of simulation time, running the simulation 4 times each. When running individual model runs over a 10-year period with a population of 20,000, all individual model runs produced an average incidence of RTIs which fell within the 95% uncertainty interval predicted by the GBD study. We performed 4 model runs per scenario as this managed computational time whilst producing results which consistently fell within our calibration targets (see ‘Appendix’).

### Comparison between single injury and multiple injury RTI models

To investigate the effect of single and multiple injuries in other areas of population health, we compare two forms of our model: one where we only give those injured in road accidents single injuries and one where we give out multiple injuries. In both forms of the model, the incidence of RTI in the population is calibrated to the average incidence of RTI predicted by GBD for Malawi in 2010–2019. By comparing the results of the two models, we see the influence of considering multiple injuries on the health outcomes for RTIs.

### Estimating the reduction in health burden attributed to the health system

To demonstrate the usefulness of explicitly modelling the health system, we investigate the reduction in harm to population health caused by the health system. We ran the model as normal, with health care being provided by the health system, and then ran the model without health care being provided by the health system (effectively all injured persons going through the ‘didn’t seek care’ route of the model, see Fig. 1). We compared the results of each scenario to find the reduction in DALYs and deaths attributed to the health system.

### Calculating summary statistics from the model

During the course of a simulation, the model will perform a routine logging event for each month of simulated time. Within this logging event, we can calculate summary statistics of interest, for example, the number of people involved in a road traffic accident, their age/sex demographics and their health outcomes. These summary statistics are stored as a logfile, from which we can analyse the model’s behaviour over time. Based on these monthly calculations, we can calculate yearly averages for our model’s outputs, which were used in the model’s calibration and form the basis of the model’s results.

## Results

### Calibration results

**Fig. 2:**
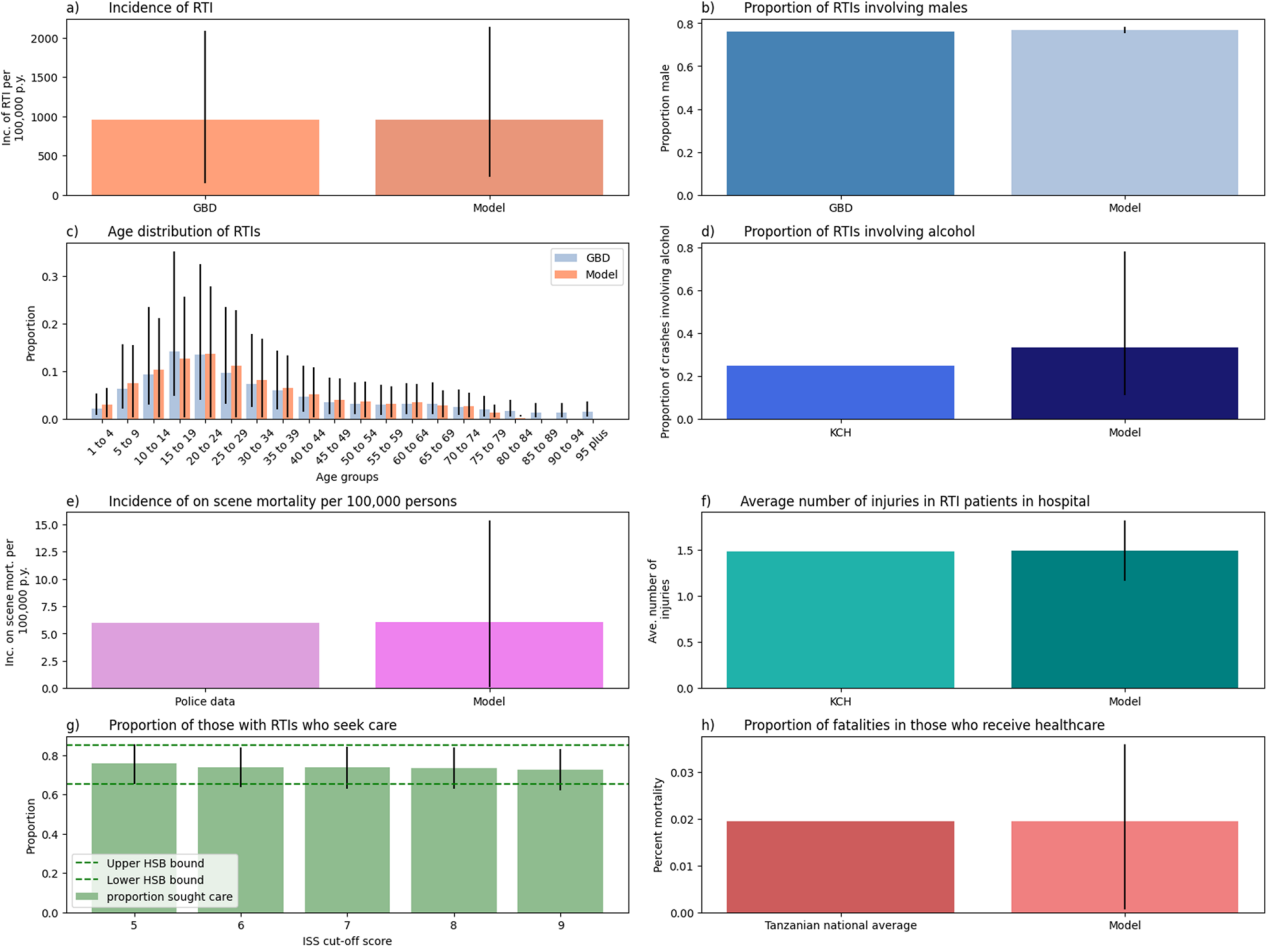
Model calibration. Panel **a** shows the calibration of the model’s incidence of RTIs to the mean incidence of RTIs predicted by the GBD study between 2010 and 2019 in Malawi. Panel **b** shows the calibration of the model’s predicted proportion of RTIs involving males to the average proportion of males from the GBD estimates 2010–2019. Panel **c** shows the age distribution of those involved in RTIs. Panel **d** shows the proportion of RTIs involving alcohol. Panel **e** shows the calibration of the model’s predicted incidence of on-scene mortality to an estimate derived from Malawi’s police data (Schlottmann et al. 2017). Panel **f** shows the model’s predicted average number of injuries per person calibrated to results from Kamuzu Central Hospital (KCH) (Sundet et al. 2018). Panel **g** shows the calibration effort to produce HSB falling within the bounds reported in other SSA countries (Zafar et al. 2018). Finally, panel **h** shows the calibration of the model’s overall predicted mortality of those who have received treatment, taking the relationship between ISS scores and mortality reported by Kuwabara et al. (2010) and scaling this to the results from a national-scale study on mortality with treatment in Tanzania (Sawe et al. 2021)

The calibration of the model managed to successfully reproduce many of the model’s target outputs, Fig. 2a shows the calibration of the overall incidence of RTIs in the population to the GBD estimate, Fig. 2b shows the calibration of the overall percentage of RTIs that involve males to compared to the GBD estimate, Fig. 2c shows the calibration of the age distribution for those with RTIs in the population to the GBD estimates, Fig. 2d shows the model’s resulting percentage of RTIs that involve alcohol consumption compared to output from Kamuzu Central Hospital (Sundet et al. 2020), Fig. 2e shows the calibration of the overall incidence of on-scene mortality RTIs in the population to the estimate from Malawi police data (Schlottmann et al. 2017), Fig. 2f shows the calibration number of injuries of those in the health system, to an estimate from Kamuzu Central Hospital (Sundet et al. 2018), Fig. 2g shows the calibration of the overall percentage of HSB, compared to the bounds reported in other SSA countries (Zafar et al. 2018), and Fig. 2h shows the calibration percentage of overall in-hospital mortality to the Tanzanian national average in-hospital mortality for RTIs (Sawe et al. 2021). Based on the range of parameters which produced HSB that fit within the ranges reported by Zafar et al. (2018) (Fig. 2g), we recalibrated several model parameters to produce the above calibration targets for each value of rt_emergency_care_ISS_score_cut_off to ensure full calibration in each model run (Table 4).

## A comparison of our model to the GBD estimates of health burden caused by RTIs in Malawi

### Incidence of mortality

**Fig. 3:**
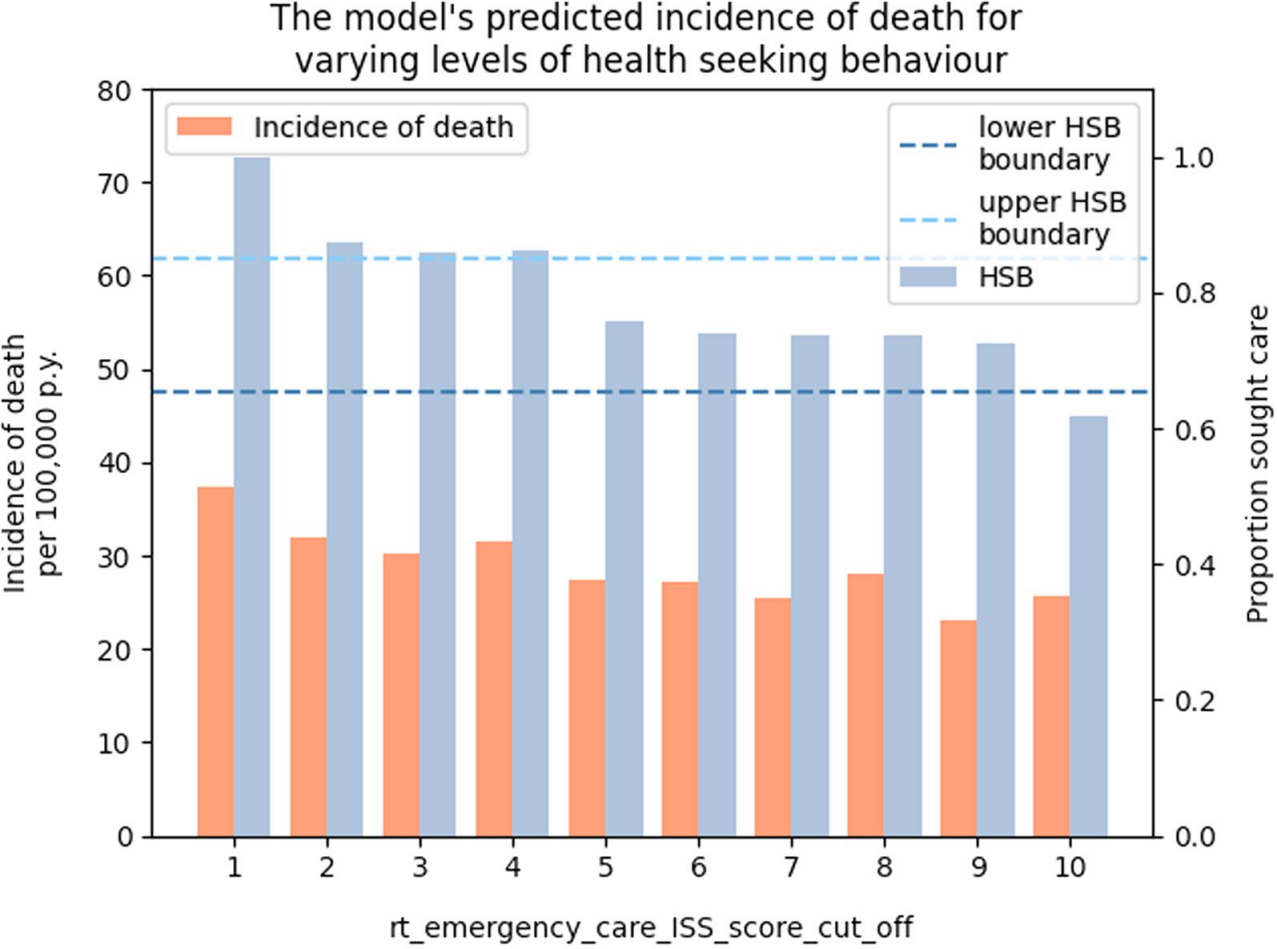
The multiple injury model’s predicted incidences of death for different values of rt_emergency_care_ISS_score_cut_off. We find that the values 5 to 9 of the parameter rt_emergency_care_ISS_score_cut_off produce levels of HSB which fall within the bounds reported by Zafar et al. (2018) and conclude the model’s estimated incidence of mortality ranges from 23.5 to 29.8 per 100,000 person years

Based on our calibration to the GBD’s estimate for the incidence of RTIs in Malawi, our estimated incidence of mortality ranged from 23.5 to 29.8 per 100,000 person years, depending on the overall percentage of people seeking care for their injuries (Fig. 3).

### Disability

**Fig. 4:**
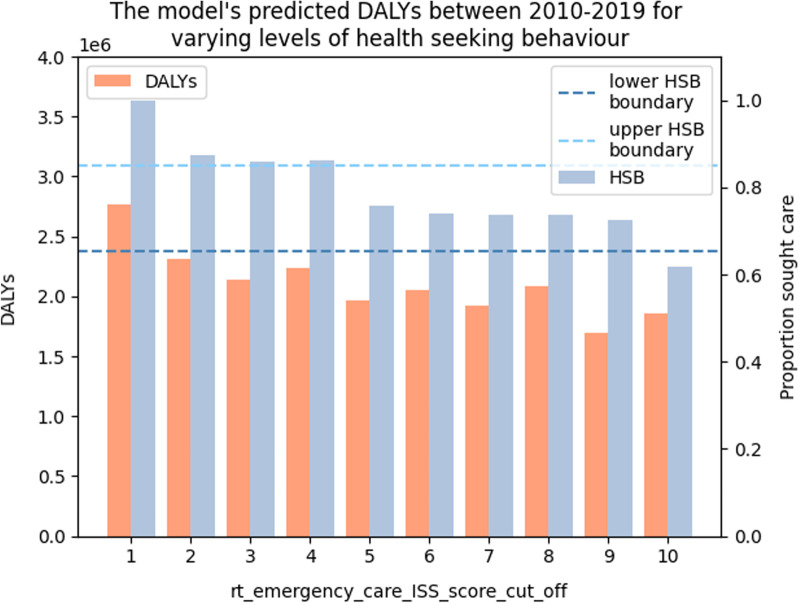
The model's predicted total number of DALYs caused by RTIs between 2010 and 2019 for varying values of rt_emergency_care_ISS_score_cut_off. The values of rt_emergency_care_ISS_score_cut_off between 5 and 9 produce an overall percentage of HSB which falls within the ranges reported by Zafar et al. (2018). From these runs, we conclude that the model predicts roughly 1.8 to 2.3 million DALYs occurring as a result of RTIs

Based on our calibration to the GBD’s estimated incidence of RTIs, our model predicts between 1,800,000 and 2,250,000 DALYs caused by RTIs from 2010 to 2019 (Fig. 4).

### Comparing the multiple injury and single injury forms of the model

**Fig. 5:**
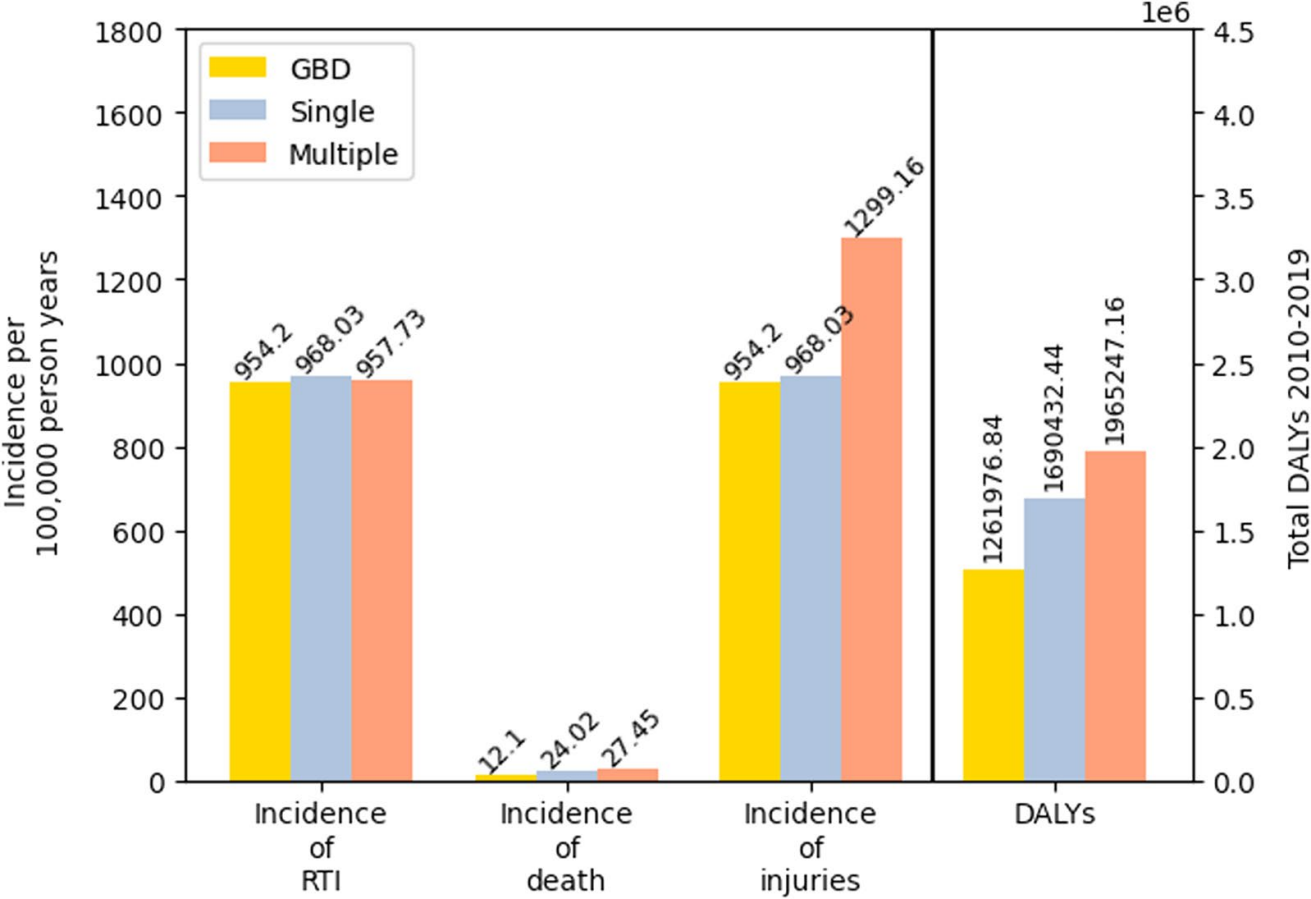
A comparison of the GBD RTI model, the single injury model and the multiple injury model. Both the single and multiple injury model predicts a higher health burden caused by RTIs than the GBD study, with both predicting a higher incidence of mortality and a greater number of DALYs. By comparing the single and multiple injury forms of the model, we see that accounting for multiple injuries led to a roughly 45% increase in health burden caused by RTIs

As in the multiple injury form of the model, the overall health burden predicted by the models was dependent on the percentage of people who sought health care for their injuries. We compared the results of the single and multiple injury models for the values of the parameter ‘rt_emergency_care_ISS_score_cut_off’ which produced HSB which fell within the ranges reported in Zafar et al. (2018). In the single injury form of the model, the average incidence of mortality per 100,000 person years ranged from 18.8 to 21.3 for each parameter value of ‘rt_emergency_care_ISS_score_cut_off’. The number of DALYs caused by RTIs from 2010 to 2019 predicted by the model ranged from 1,400,000 to 1,560,000. In both deaths and DALYs, accounting for multiple injuries resulted in a 22–58% increase to DALYs and a 20–56% increase in the number of deaths. A comparison of the results of the GBD, single and multiple injury models for ‘rt_emergency_care_ISS_score_cut_off’ = 5 is given in Fig. 5.

### The predicted effectiveness of the health system in reducing death and disability

**Fig. 6:**
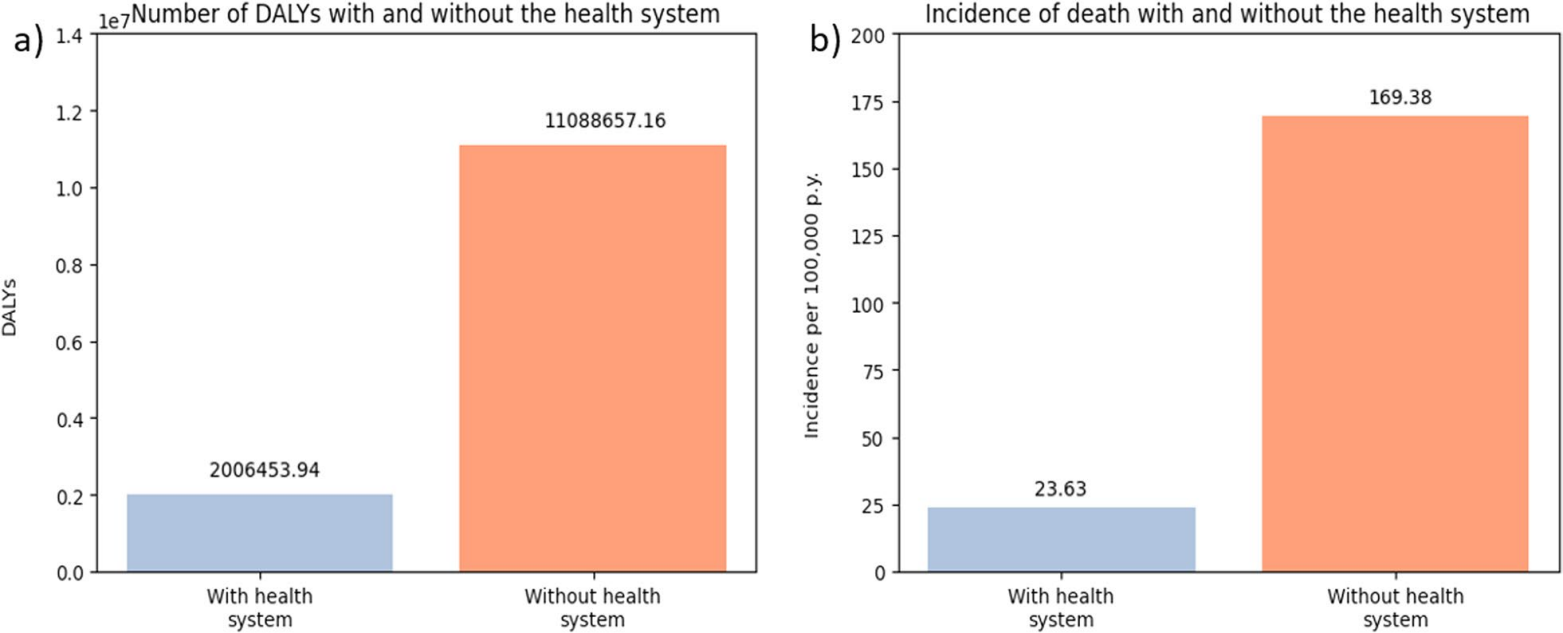
A comparison of the health burden predicted by the model with and without the health system, **a** compares the predicted number of DALYs and **b** compares the predicted incidence of mortality

Comparing model runs where the health system is allowed to provide care to runs where no health care is provided reveals the predicted effectiveness of the health system in preventing death and disability. Our model estimates an incidence of mortality ranging from 23.5 to 29.8 per 100,000 person years with the health system running. Without the health system, the model predicts an incidence of mortality of 63.15 per 100,000 person years. For DALYs, our model predicted between 1,800,000 and 2,250,000 from 2010 to 2019 with the health system and roughly 4.3 million DALYs without the health system (Fig. 6).

## Discussion

### The health burden of road traffic injuries in Malawi

Epidemiological models of RTIs are uncommon compared to other disease areas. To better understand the health burden imposed by RTIs in Malawi, we develop an IBM to estimate the incidence of mortality due to RTIs in Malawi. We linked RTI epidemiology with HSB and health system availability to form a complete health journey for individuals with RTIs, from initial injury through to recovery, disability or mortality. We calibrated our overall incidence of RTIs to the average of the GBD’s estimated incidence of RTIs between 2010 and 2019, but apart from that the assumptions and methodology between the two studies vary. We compare the predicted health burden produced by the two models.

Our model produced a higher estimated incidence of mortality compared to the GBD study, predicting between 25.95 and 28.38 deaths per 100,000 person years compared to the GBD estimate of 12.1 per 100,000 person years, meaning that our model estimates a higher case fatality ratio. Results from Nigerian population surveys that estimate 3.9% of RTIs were fatal (Bun 2012). Our model that estimates between 2.5 and 3.1% of RTIs were fatal, whereas the GBD estimates that predict 1.3% of RTIs were fatal. One of the key differences between the models is our model’s consideration of multiple injury. We conducted an internal comparison of our model as a single injury model and as a multiple injury model. The estimated incidence of mortality produced by our single injury model ranged from 18.8 to 21.3 per 100,000 person years, implying that accounting for multiple injuries in a model will increase mortality by around 5–9 deaths per 100,000 person years (roughly 22–58% increase). Even without considering the role of multiple injury and HSB, our estimated incidence of mortality in the single injury model is higher than that of the GBD study. This is likely due to our effort to calibrate in-hospital mortality to the results of Kuwabara et al. (2010), who reported a national-scale in-hospital mortality of 1.8%. As our calibration estimate for the overall level of in-hospital mortality was higher than the overall level of mortality reported by the GBD, if we assume that those who don’t seek health care would be at a higher risk of mortality than those who don’t, then the differences the number of deaths and DALYs predicted by our model can be explained by the differences in the assumptions made with respect to in-hospital mortality.

Our estimated incidence of mortality is notably higher than the GBD estimate and would indicate a much higher health burden brought about in the population of Malawi from RTIs. Other estimates for the incidence of mortality from RTIs in Malawi are also higher than the GBD estimate, such as the WHO model (WHO 2015) and the results of Samuel et al. (2012). A recent household survey in Malawi study estimated 17 RTI deaths per 100,000 person years (National Statistical Office 2021) (see calculation in ‘Appendix’).

The GBD estimated incidence of mortality is comparatively low compared to other estimates, such as the WHO’s estimate. The methodology used for the WHO’s model was similar, a country-specific regression model considering broadly similar covariates (alcohol, speed limit laws, vehicle type, health care access, population and economic income) with other additional covariates considered in each model. Given the broadly similar factors considered in predicting mortality between the GBD and WHO models, the differences in predicted incidence of mortality are surprising. One explanation for the GBD estimates being lower than both our model and the WHO’s model is the usage of police and hospital data to fit their estimated incidence of mortality. Malawi’s police and hospital records have been shown to not record every fatality (Samuel et al. 2012). Therefore, by calibrating the model estimates based on these data sources, the GBD study would be likely underestimating the extent of the deaths caused by RTIs in Malawi.

Our model predicted a greater health burden cause by RTIs as is reflected in our model’s predicted number of DALYs (Fig. 4), which predicts roughly 2 million DALYs compared to the 1.3 million predicted by the GBD study. In our internal comparison, we find that accounting for multiple injuries results in a 22–58% increase in the number of DALYs predicted in the model. Given the similarities in our assumptions, we would expect the single injury form of the model to produce a similar number of DALYs as the GBD estimate. However, owing to the greater number of deaths predicted in both the single and multiple injury forms of our model, our model predicts a greater number of DALYs than the GBD model (Fig. 5). The increased number of DALYs predicted by our model implies that there may be a greater health burden caused by RTIs in Malawi than previously thought.

One issue we faced was the uncertainty with respect to the percentage of people who sought care. We tried to account for this uncertainty by running the model for varying parameter values which produced an overall percentage of HSB falling within the range reported by Zafar et al. (2018). In each of these runs, the number of injuries and probability of death in hospital was adjusted to still match the results of studies from KCH (Tyson et al. 2015; Sundet et al. 2018), a consequence of this calibration effort was in different model runs, and a person with the same injuries would have a slightly different likelihood of mortality. This run-specific calibration was necessary as there wasn’t a Malawi-specific estimate for HSB, but without a set point to calibrate to, we were limited in our investigation of the interaction between road traffic injury epidemiology, HSB and health system usage.

Both the model’s estimated incidence of mortality and number of DALYs are dependent on the overall incidence of RTIs in the population. We calibrated this to the 10-year average incidence of RTIs estimated by the GBD study. The overall level of incidence reported by the GBD study was based on the 2003 World Health Survey in Malawi (WHO 2005), producing an estimate of the incidence of injuries caused by road traffic crashes which would warrant some form of health care. The 2003 World Health Survey for Malawi produced an estimated incidence of 3562 RTIs per 100,000, of which we estimate 954.2 RTIs per 100,000 warrant some form of health care. This shared assumption between our model and the GBD model neglects to predict the full health burden that could be caused by RTIs in Malawi. We believe our model offers some improvement to the GBD model’s estimates but without data to measure the health burden of all those with RTIs. We cannot truly estimate the exact scale of the health burden caused by RTIs, but believe that we account for the vast majority of health burden with this model.

### Modelling discussion

Building a model incorporating as much detail as has been included in this model requires a lot of data driven parameter estimates in the epidemiological, HSB and health system components of the model. Where available, we used appropriate parameters from Malawi; for example, the HSB model is based entirely on Malawian survey data (Ng’ambi et al. 2020a, 2020b) and the health system model is based on Malawi’s treatment guidelines (Ministry of Health 2015), the Clinton Health Access Initiative (CHAI) and EHP datasets (Government of Malawi 2018). In Malawi, the majority of the published RTI literature comes from central hospitals, some of the countries’ best equipped hospitals. Owing to the facilities available at this hospital, many severely injured patients are transferred from other hospitals (e.g. 60.4% of traumatic brain injury patients (Eaton et al. 2017) and 66.7% of spinal cord injury patients (Eaton et al. 2019)). This may imply that the parameters used to describe certain aspects of the model, such as the number of injuries assigned per person, would be calibrated to represent severely injured patients rather than the average injured person; however, the percentage of multiple injuries predicted by the model do fall within ranges reported from other SSA A&E departments (Akinpelu et al. 2007; Ganveer and Tiwari 2005; Madubueze et al. 2010; Sanyang et al. 2017; Thanni and Kehinde 2006). When a Malawi-specific parameter was not available, we had to look further afield to inform our parameter estimates. In such cases, parameters based on reports from other Sub-Saharan African countries were preferred, but in some instances, we had to look for data outside of the continent. The results of this model are heavily dependent on the parameters we use to make our predictions. We run the risk of assuming that the parameters used in the model are the only set of parameters which produce reasonable results.

In IBM, the model’s behaviour is established through running the model. This approach inherently produces stochasticity between runs, so the result of any particular run is unique to that run and may not truly accurately represent the model’s overall behaviour. To overcome this, there are two effective strategies. The first is to increase the number of individuals in the system (effectively increasing the sample size); the second approach is to repeatedly run simulations and take an average of the results of all runs. Both strategies to account for stochasticity result in increased computational time. To optimise our approach, we investigated the effect of running larger population sizes vs repeated model runs in an attempt to reduce the standard error of our model’s results in the most efficient way possible. We ran the model with a large model population size of 1 million and then repeatedly sampled the model population in increasing increments of population size, calculating summary statistics relevant to RTI epidemiology. We find that a smaller model population size of around 20,000 individuals with repeated samples provided an adequate representation of a larger model population (see TLO model website) and accounts for a large part of the model’s stochasticity.

The results of our model as in the case of all models are dependent on the assumptions that we have made. For example, the estimate for the incidence of mortality produced by our model is dependent on the estimated incidence of RTIs predicted in the first place, which we calibrated to the GBD study’s estimated average incidence over the ten-year period. The GBD estimates were the only population wide estimate for the incidence of RTIs in Malawi that also provided an estimate for the incidence of mortality; as such, the results of our model are dependent on the validity of the GBD estimates. We also based our model’s predicted number of injuries on results from a study focusing on paediatric patients (Sundet et al. 2018). There is limited published data on the number of injuries caused by RTIs per person in Malawi, and age does not appear to be a significant predictor of the number of injuries received in road accidents (Nasrullah and Muazzam 2012). Another source of uncertainty in our estimates comes from our assumptions of the probability of death without medical intervention. The only reasonably appropriate estimate which could be used to represent the likelihood of death from injury without adequate medical care came from a study on military-related injuries (Champion et al. 2010). The inevitable limited availability of data to inform this section of the model makes the comparison of population health burden with and without the health system speculative. The results of the health system vs no health system comparison present the worst-case scenario RTIs in Malawi, predicting 63.15 deaths per 100,000 person years.

In our model, we made simplifying assumptions and chose not to include many links between the physical world and road traffic injury epidemiology. We did not consider physical factors such as vehicle speed, road vehicle type and the role of each person in a crash (pedestrian, cyclist, motorcyclist, etc.). These amongst several other physical factors influence the severity of a crash and the health outcomes that follow (Ranti et al. 2015). The effect of certain spatial and temporal fluctuations is considered in some parts of the TLO model. For example, each simulated person belongs to a district which will determine certain demographic factors about each person. The care the health system provides is also spatially dependent, with treatment availability fluctuating over time for each facility within each district (a more detailed description of the demography and health system models can be found on the documentation section of the TLO model website: https://www.tlomodel.org/writeups.html). As the road traffic injury model makes use of the TLO demography and health system models, there is some inherited spatial and temporal fluctuations the population modelled and the treatment of injuries. However, we do not consider the effect of time or space in the occurrence of road traffic injuries. This assumption was made in an attempt to simplify the model where possible, whilst still capturing national average trends over the ten-year period of study. Another limitation to this model’s approach is the length of the time the model takes between updating changes to the population and health system availability and usage. The smallest increment of time considered in the TLO model is 24 h, meaning that we are unable to capture the effect of short-term delays in receiving emergency care on health outcomes.

Unlike communicable and non-communicable health conditions, there is no causal organism or other biological aspect that causes RTIs; instead abiotic factors such as vehicle type, traffic law enforcement, road surface and weather amongst others will influence the number of injurious road accidents and the health burden caused by these crashes. In the model presented here, we explicitly model population demographics, population lifestyle, HSB and health care availability. This RTIs model is designed to work in line with other disease models, all designed with their own epidemiological nuances and details to represent their diseases in the context of Malawi. As such, unlike the WHO’s and the GBD’s models discussed in this paper, we do not explicitly model aspects of the physical world that are included in other models of RTIs in Malawi such as traffic legislation, vehicle number and vehicle type.

In this paper, we show the strength of an IBM approach for modelling the link between epidemiology, HSB and health system usage. IBMs more easily allow for complex layering of modelling detail than other modelling approaches at the expense of explicit solutions. By incorporating greater levels of detail in the model, such as additional HSB and the model health system, we can investigate the interaction between components, as was demonstrated in the single vs multiple injury model comparison. In the single vs multiple injury example, we manipulated the epidemiology of RTIs; however, this is just an example of what is possible with the model. We can manipulate any of the modelling components, making changes to the road traffic injury, HSB and health system models to explore the effects of changes to the system.

Through developing the model, we noticed a number of knowledge gaps in the road traffic injury research area. The first issue is that there are limited estimates for the incidence of RTIs and road traffic injury deaths occurring in Malawi which aren’t based on models. The model-based estimates of incidence vary greatly between sources, with the GBD predicting an average incidence of 954.2 per 100,000 person years from 2020 to 2019 and the World Health Survey predicting an incidence of 3562 per 100,000 person years. The GBD study based their estimates incidence of RTIs on the World Health Survey estimate, only accounting for those injured persons whose injuries would require treatment. Given that many injuries will go unreported, we have a limit in our ability to truly understand the full scale of the road traffic injury epidemic in Malawi and in other countries. Unreported injuries also result in a potentially skewed image of what the typical injuries that are experience by someone involved in a road accident. Based on the currently available information, this model and the GBD model can use information from hospital A&E reports to predict the health burden caused by RTIs, but because this information is taken from a subsection of all RTIs, any predictions on the health burden caused by RTIs are extrapolations. This is also an issue for estimating the health burden of injuries in general. What we believe would be a useful study to truly get understand the full extent of the health burden caused by injuries in Malawi and elsewhere would be a household survey, which gathered information on the number of people involved in RTIs, the number of people who have died as a result of RTIs and information relevant to the exact nature of injuries received from collisions, such as the number of injuries, the type of injuries and the anatomic location of injuries. Such a study would provide a clearer image of the health burden caused by RTIs. Another general need is for complete death registration with cause. Whilst death registration is mandated by Malawian law, there are known gaps in the recording of deaths (Singogo et al. 2013).

The overall Thanzi La Onse model is built with a modular disease epidemiology design. Just as we manipulated HSB by including and not including the HSB model, we can manipulate the model by including other disease modules and simultaneously examine how several diseases interact with and compete with the health system. The inclusion of other modules will allow a full picture of Malawi’s population health which will be useful in planning public health expenditure.

## Conclusions

This paper introduces a novel mathematical modelling framework for modelling road traffic injuries with a level of detail that had previously not been achieved. We used this model to produce an estimated number of deaths and DALYs caused by RTIs in Malawi, finding that estimated 23.5–29.8 deaths occur per 100,000 person years and estimated 180,000–225,000 DALYs occurred per year in an estimated population of 1,364,000 in Malawi.

## Figures and Tables

**Fig. 7:**
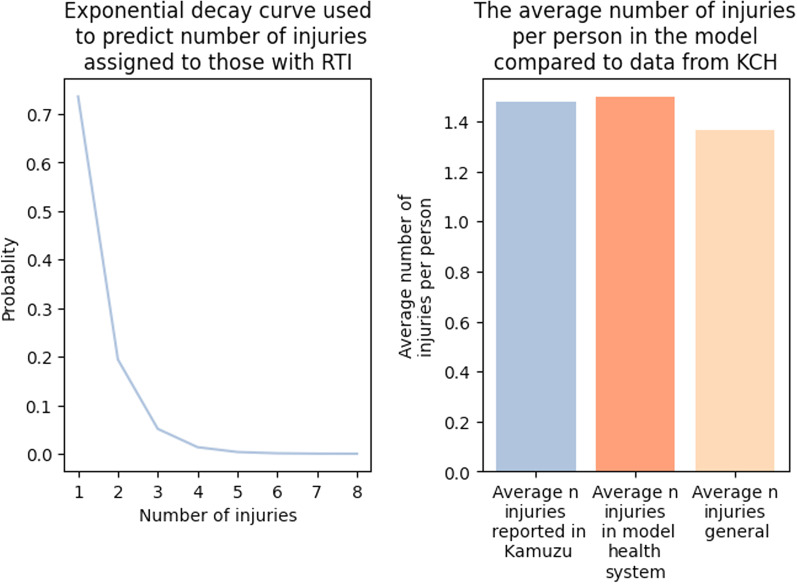
An example distribution of number of injuries assigned to each per person and the resulting average number of injuries per person resulting from the distribution compared to the results of Sundet et al. (2018)

**Fig. 8:**
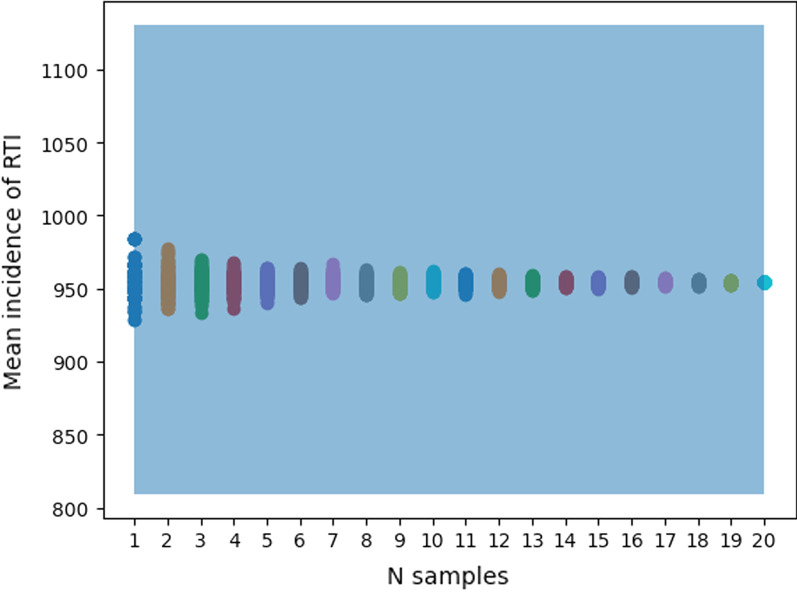
The effect on the number of model runs on the average incidence of road traffic injuries in the model. For each number of samples (*x*-axis), we sampled *N* runs from the 20 model runs 100 times. We then plotted the average incidence of road traffic injuries between the N model runs as circles, associated with the number of runs sampled per time. We then plotted the 95% uncertainty interval of the incidence of road traffic injuries between 2010 and 2019 taken from the GBD study (shown in blue). We find that all of the 20 model runs fell within the 95% uncertainty interval of incidence of road traffic injuries within the GBD study

## Data Availability

Reference material and code can be found on the TLO model website: https://www.tlomodel.org/

## Appendix

### Calculating estimates of incidence of road traffic injuries and road traffic injury deaths from survey data

#### Incidence of road traffic injuries, World Health Survey

The sample size of this study was 5297, of which 3.5% were injured in a road accident, with rounding this indicates 185 people were injured over the course of the study (WHO 2005). The study questioned participants about injuries in the last year; if we assume that on average each injury occurred 6 months prior to question, we establish our estimate as (Table 1):

$${\text{Incidence}}\;{\text{of}}\;{\text{RTI}}\;{\text{person}}\;{\text{year}} = \frac{{185}}{{5297 - 0.5 \times 185}} = 0.03562$$

$${\text{Incidence}}\;{\text{of}}\;{\text{RTI }}100,000\;{\text{person}}\;{\text{years}} = 3562$$

**Table 1:** Properties modelled by the road traffic injuries model

Properties modelled	
Property name	Description
rt_road_traffic_inc	Whether this person has been injured in a road traffic crash
rt_date_inj	The date which this person was injured in a road accident
rt_date_death_no_med	The date which this person’s mortality is determined if they haven’t sought care, assumed to be a week after their accident
rt_diagnosed	Whether this person has sought care at a hospital and can progress further into the health system
rt_med_int	Whether this person is receiving care for their injuries
rt_in_icu_or_hdu	Whether this person is in an intensive care or high dependency unit
rt_date_to_remove_daly	The dates in which each of this person’s injuries will heal and the DALY weight associated with each injury can be removed
rt_in_shock	Whether this person is in shock as a result of their injuries
rt_polytrauma	Whether this person has two injuries in distinct body regions with an AIS score of 3 or greater
rt_perm_disability	Whether this person is permanently disabled as a result of their injuries
rt_recovery_no_med	Whether this person’s injuries healed without the use of the health system
rt_injury_1, rt_injury_2, rt_injury_3, rt_injury_4, rt_injury_5, rt_injury_6, rt_injury_7, rt_injury_8,	The injuries this person received from their road accident, with the above injuries being stored as codes in these properties
rt_injury_severity	The severity status of this person’s injuries, none, mild or severe
rt_ISS_score	The injury severity score of this person
rt_MAIS_military_score	The maximum military abbreviated injury score associated with this person’s injuries
rt_disability	The DALY weight associated with this person’s injuries, capped between 0 and 1
rt_debugging_DALY_wt	The true DALY weight associated with this person’s injuries, uncapped
rt_injuries_to_cast	Injuries of this person determined to be treated by fracture cast or sling
rt_injuries_for_minor_surgery	Injuries of this person determined to be treated with a minor surgery
rt_injuries_for_minor_surgery	Injuries of this person determined to be treated with a major surgery
rt_injuries_for_open_fracture_treatment	Injuries of this person determined to be treated with an open fracture treatment plan
rt_injuries_to_heal_with_time	Injuries which will heal over time without specific interventions
rt_injuries_left_untreated	The person’s injuries that have been left untreated
rt_imm_death	Whether this person died on the scene of the crash or not
rt_death_no_med	Whether this person died without seeking medical care
rt_post_med_death	Whether this person died from their injuries despite medical intervention
rt_unavilable_med_death	Whether this person died after seeking medical care due to the unavailability of certain treatments
rt_death_from_shock	Whether this person died from shock

These properties were used to keep track of road traffic injuries in the population, the health system usage and health outcomes

#### Incidence of road traffic injury death, integrated household survey

The data for this survey were collected by asking a member of the household about their and other people in the household’s health experience in the past 2 years (National Statistical Office 2021). This data also established the number of people in each household. This survey found 17 road traffic injury deaths within the households surveyed over the past two years. At the time of the survey, a total of 50,476 people had been accounted for in the survey, either directly reporting to the interviewers or having their past health experiences accounted for by a member of the household. If we again assume that on average the road traffic injury deaths occurred halfway through the study (in this case a two-year study), we can establish an estimate for the incidence of mortality as (Table 2):

$${\text{Incidence}}\;{\text{of}}\;{\text{RTI}}\;{\text{death}}\;{\text{per}}\;{\text{person}}\;{\text{year}} = \frac{{17}}{{50476 \times 2 - 17 \times 1}} = 0.000168$$

$${\text{Incidence}}\;{\text{of}}\;{\text{RTI}}\;{\text{death}}\;{\text{per}}\;100,000\;{\text{person}}\;{\text{years}} = 16.8$$

**Table 2:** Parameters used in the model, with name, description, value and source/calibration process

Parameter	Description	Value		Source
*Who is injured in a road traffic crash this month?*				
Base_rate_injrti	The base rate of which the model population is involved in road traffic collisions per month. Specifically, a woman above the age of 80 who doesn’t drink	Run-specific, see Table 4		Calibrated to GBD estimated incidence of RTI
rr_injrti_age04	The risk factor for having a road traffic injury associated with being aged 0–4	0.145533		Calibrated to GBD estimated incidence of RTI
rr_injrti_age59	The risk factor for having a road traffic injury associated with being aged 5–9	0.551895		Calibrated to GBD estimated incidence of RTI
rr_injrti_age1017	The risk factor for having a road traffic injury associated with being aged 10–17	0.967017		Calibrated to GBD estimated incidence of RTI
rr_injrti_age1829	The risk factor for having a road traffic injury associated with being aged 18–29	1.184184		Calibrated to GBD estimated incidence of RTI
rr_injrti_age3039	The risk factor for having a road traffic injury associated with being aged 30–39	1.052843		Calibrated to GBD estimated incidence of RTI
rr_injrti_age4049	The risk factor for having a road traffic injury associated with being aged 40–49	1.074376		Calibrated to GBD estimated incidence of RTI
rr_injrti_age5059	The risk factor for having a road traffic injury associated with being aged 50–59	1.336449		Calibrated to GBD estimated incidence of RTI
rr_injrti_age6069	The risk factor for having a road traffic injury associated with being aged 60–69	2.308514		Calibrated to GBD estimated incidence of RTI
rr_injrti_age7079	The risk factor for having a road traffic injury associated with being aged 70–79	4.031226		Calibrated to GBD estimated incidence of RTI
rr_injrti_male	The risk factor for having a road traffic injury associated with being male	2.7		Calibrated to GBD estimated incidence of RTI
rr_injrti_excessalcohol	The risk factor for having a road traffic injury associated with consuming excessive amounts of alcohol	6.53		Staton et al. (2018)
*Did the injured persons die on the scene of the crash*				
imm_death_proportion_rti	The proportion of persons injured in a road traffic collision who experience pre-hospital mortality	0.018		Mulima et al. (2021)
*What injuries did each injured person receive?*				
number_of_injured_body_regions_distribution	The distribution used to assign the number of injured body regions for each injured person	Run-specific, see Table 4		Distributions calibrated to the results of Sundet et al. (2018)
injury_location_distribution	The distribution used to assign the anatomic location of the person’s injuries	Location	Probability	Ranti et al. (2015), Otieno et al. (2004)
		Head	14.38	
		Face	13.25	
		Neck	2.1	
		Thorax	9.45	
		Abdomen	6.12	
		Spine	1.55	
		Upper Extremity	16.85	
		Lower Extremity	36.3	
head_prob_112	Probability of an unspecified skull fracture	0.0455		Eaton et al. (2017), Global Health Data (2017)
head_prob_113	Probability of a basilar skull fracture	0.0045		Eaton et al. (2017), Global Health Data (2017)
head_prob_133a	Probability of a subarachnoid hematoma	0.09149906		Carroll et al. (2010), Global Health Data (2017), Eaton et al. (2017)
head_prob_133b	Probability of a brain contusion	0.301946898		Carroll et al. (2010), Global Health Data (2017), Eaton et al. (2017)
head_prob_133c	Probability of an intraventricular haemorrhage	0.013724859		Carroll et al. (2010), Global Health Data (2017), Eaton et al. (2017)
head_prob_133d	Probability of a subgaleal hematoma	0.050324483		Carroll et al. (2010), Global Health Data (2017), Eaton et al. (2017)
head_prob_134a	Probability of an epidural hematoma	0.086670324		Carroll et al. (2010), Global Health Data (2017), Eaton et al. (2017)
head_prob_134b	Probability of a subdural hematoma	0.080003376		Carroll et al. (2010), Global Health Data (2017), Eaton et al. (2017)
head_prob_135	Probability of a diffuse axonal injury/midline shift	0.061731		Carroll et al. (2010), Global Health Data (2017), Eaton et al. (2017)
head_prob_1101	Probability of a laceration to the head	0.253536		Malm et al. (2008), Global Health Data (2017)
head_prob_1114	Probability of a burn to the head	0.010564		Tian et al. (2018), Global Health Data (2017)
face_prob_211	Probability of a facial fracture (nasal/unspecified)	0.158585		Hassan (2016)
face_prob_212	Probability of a facial fracture (mandible/zygomatic)	0.294515		Hassan (2016)
face_prob_241	Probability of a soft tissue injury to face	0.339		Hassan (2016)
face_prob_2101	Probability of a laceration to the face	0.194845		Malm et al. (2008), Global Health Data (2017)
face_prob_2114	Probability of a burn to the face	0.010255		Tian et al. (2018), Global Health Data (2017)
face_prob_291	Probability of an eye injury	0.0028		Hassan (2016)
neck_prob_3101	Probability of a laceration to the neck	0.06972		Malm et al. (2008), Global Health Data (2017)
neck_prob_3113	Probability of a burn to the neck	0.01428		Tian et al. (2018), Global Health Data (2017)
neck_prob_342	Probability of a soft tissue injury in neck (vertebral artery laceration)	0.004		Kasantikul et al. (2003)
neck_prob_343	Probability of a soft tissue injury in neck (pharynx contusion)	0.004		Kasantikul et al. (2003)
neck_prob_361	Probability of a Sternomastoid m. haemorrhage/Haemorrhage, supraclavicular triangle/Haemorrhage, posterior triangle/Anterior vertebral vessel haemorrhage/Neck muscle haemorrhage	0.495		Kasantikul et al. (2003)
neck_prob_363	Probability of a Hematoma in carotid sheath/Carotid sheath haemorrhage	0.405		Kasantikul et al. (2003)
neck_prob_322	Probability of an atlanto-occipital subluxation	0.00264		Kasantikul et al. (2003)
neck_prob_323	Probability of an atlanto-axial subluxation	0.00536		Kasantikul et al. (2003)
thorax_prob_4101	Probability of a laceration to the thorax	0.49036		Malm et al. (2008), Global Health Data (2017)
thorax_prob_4113	Probability of a burn to the thorax	0.04264		Tian et al. (2018), Global Health Data (2017)
thorax_prob_461	Probability of chest wall bruises/haematoma	0.0945		Okugbo et al. (2012)
thorax_prob_463	Probability of haemothorax	0.0945		Okugbo et al. (2012)
thorax_prob_453a	Probability of a lung contusion	0.0539		Okugbo et al. (2012)
thorax_prob_453b	Probability of a diaphragm rupture	0.0161		Okugbo et al. (2012)
thorax_prob_412	Probability of fractured ribs	0.0392		Okugbo et al. (2012)
thorax_prob_414	Probability of flail chest	0.0098		Okugbo et al. (2012)
thorax_prob_441	Probability of chest wall lacerations/avulsions	0.08586		Okugbo et al. (2012)
thorax_prob_442	Probability of surgical emphysema	0.01749		Okugbo et al. (2012)
thorax_prob_443	Probability of closed pneumothorax/open pneumothorax	0.05565		Okugbo et al. (2012)
abdomen_prob_5101	Probability of a laceration to the abdomen	0.11026		Malm et al. (2008), Global Health Data (2017)
abdomen_prob_5113	Probability of a burn to the abdomen	0.03874		Tian et al. (2018), Global Health Data (2017)
abdomen_prob_552	Probability of an injury to stomach/intestines/colon	0.047656		Global Health Data (2017), Ruhinda et al. (2008)
abdomen_prob_553	Probability of injury to spleen/Urinary bladder/Liver/Urethra/Diaphragm	0.77441		Global Health Data (2017), Ruhinda et al. (2008)
abdomen_prob_554	Probability of an injury to kidney	0.028934		Global Health Data (2017), Ruhinda et al. (2008)
spine_prob_612	Probability of fractured vertebrae	0.364		Biluts et al. (2015)
spine_prob_673a	Probability of a spinal cord injury at neck level with an AIS score of 3	0.015840216		Biluts et al. (2015), Stephan et al. (2015)
spine_prob_673b	Probability of a spinal cord injury below neck level with an AIS score of 3	0.040731984		Biluts et al. (2015), Stephan et al. (2015)
spine_prob_674a	Probability of a spinal cord injury at neck level with an AIS score of 4	0.074477731		Biluts et al. (2015), Stephan et al. (2015)
spine_prob_674b	Probability of a spinal cord injury below neck level with an AIS score of 4	0.116490809		Biluts et al. (2015), Stephan et al. (2015)
spine_prob_675a	Probability of a spinal cord injury at neck level with an AIS score of 5	0.134791137		Biluts et al. (2015), Stephan et al. (2015)
spine_prob_675b	Probability of a spinal cord injury below neck level with an AIS score of 5	0.210827163		Biluts et al. (2015), Stephan et al. (2015)
spine_prob_676	Probability of a spinal cord injury at neck level with an AIS score of 6	0.04284096		Biluts et al. (2015), Stephan et al. (2015)
upper_ex_prob_7101	Probability of a laceration to the upper extremities	0.43896		Malm et al. (2008), Global Health Data (2017)
upper_ex_prob_7113	Probability of a burn to the upper extremities	0.03304		Tian et al. (2018), Global Health Data (2017)
upper_ex_prob_712a	Probability of a fracture to Clavicle, scapula, humerus	0.10802		Global Health Data (2017)
upper_ex_prob_712b	Probability of a fracture to Hand/wrist	0.28969		Global Health Data (2017)
upper_ex_prob_712c	Probability of a fracture to Radius/ulna	0.09329		Global Health Data (2017)
upper_ex_prob_722	Probability of a dislocated shoulder	0.025		Global Health Data (2017)
upper_ex_prob_782a	Probability of an amputated finger	0.00750024		Global Health Data (2017)
upper_ex_prob_782b	Probability of a unilateral arm amputation	0.00102276		Global Health Data (2017)
upper_ex_prob_782c	Probability of a thumb amputation	0.002841		Global Health Data (2017)
upper_ex_prob_783	Probability of a bilateral upper extremity amputation	0.000636		Global Health Data (2017)
lower_ex_prob_8101	Probability of a laceration to the lower extremity	0.186094109		Malm et al. (2008), Global Health Data (2017)
lower_ex_prob_8113	Probability of a burn to the lower extremity	0.014007083		Tian et al. (2018), Global Health Data (2017)
lower_ex_prob_811	Probability of a foot fracture	0.023610948		Global Health Data (2017)
lower_ex_prob_813do	Probability of an open foot fracture	0.013281158		Global Health Data (2017), Court-Brown et al. (2012). Chagomerana et al. (2017)
lower_ex_prob_812	Probability of a fracture to patella, tibia, fibula, ankle	0.354164215		Global Health Data (2017)
lower_ex_prob_813eo	Probability of an open fracture to patella, tibia, fibula, ankle	0.199217371		Global Health Data (2017), Court-Brown et al. (2012), Chagomerana et al. (2017)
lower_ex_prob_813a	Probability of a hip fracture	0.029513685		Global Health Data (2017)
lower_ex_prob_813b	Probability of a pelvis fracture	0.023610948		Global Health Data (2017)
lower_ex_prob_813bo	Probability of an open pelvis fracture	0.005902737		Global Health Data (2017), Court-Brown et al. (2012), Chagomerana et al. (2017)
lower_ex_prob_813c	Probability of a femur fracture	0.076765094		Global Health Data (2017)
lower_ex_prob_813co	Probability of an open femur fracture	0.01177596		Global Health Data (2017), Court-Brown et al. (2012), Chagomerana et al. (2017)
lower_ex_prob_822a	Probability of a dislocated hip	0.037338982		Global Health Data (2017)
lower_ex_prob_822b	Probability of a dislocated knee	0.002383339		Global Health Data (2017)
lower_ex_prob_882	Probability of a amputation of toes	0.00731139		Global Health Data (2017)
lower_ex_prob_883	Probability of a unilateral lower leg amputation	0.007511491		Global Health Data (2017)
lower_ex_prob_884	Probability of a bilateral lower leg amputation	0.007511491		Global Health Data (2017)
*Did they go on to seek health care for their injuries?*				
rt_emergency_care_ISS_score_cut_off	The ISS score above which people will automatically go to seek health care	Run-specific, see Table 4		Calibrated to the results of Zafar et al. (2018)
*If they sought health care for their injuries, what do they need from the health system for their treatment?*				
mean_los_ISS_less_than_4	The mean length of stay for a person with an ISS score less than 4	4.97		Lee et al. (2016)
sd_los_ISS_4_to_8	Variation length of stay for those with an ISS score between 4 and 8	5.93		Lee et al. (2016)
mean_los_ISS_9_to_15	Mean length of stay for those with an ISS score between 9 and 15	15.46		Lee et al. (2016)
sd_los_ISS_9_to_15	Variation in length of stay for those with an ISS score between 9 and 15 (Lee et al. 2016)	11.16		Lee et al. (2016)
mean_los_ISS_16_to_24	Mean length of stay for those with an ISS score between 16 and 24	24.73		Lee et al. (2016)
sd_los_ISS_16_to_24	Variation in length of stay for those with an ISS score between 16 and 24	17.03,		Lee et al. (2016)
mean_los_ISS_more_than_25	Mean length of stay for those with an ISS score greater than 25	30.86		Lee et al. (2016)
sd_los_ISS_more_that_25	Variation length of stay for those with an ISS score greater than 25	34.03		Lee et al. (2016)
prob_dislocation_requires_surgery	Probability that a dislocation will require a surgery	0.01		Dummy variable used to account for the fact that some dislocations will require surgery
prob_depressed_skull_fracture	Probability that the person’s skull fracture is depressed and will require surgery	0.14		Eaton et al. (2017)
prob_open_fracture_contaminated	Probability that the person’s open fracture is contaminated	0.07		Chagomerana et al. (2017)
*Based on their choice to seek or not seek health care what health outcomes did each person experience (mortality, morbidity or recovery)?*				
prob_death_iss_less_than_9	The probability of mortality associated with an ISS score less than 9 with medical treatment	Run-specific, see Table 4		Kuwabara et al. (2010), Tyson et al. (2015)
prob_death_iss_10_15	The probability of mortality associated with an ISS score between 10 and 15 with medical treatment	Run-specific, see Table 4		Kuwabara et al. (2010), Tyson et al. (2015)
prob_death_iss_16_24	The probability of mortality associated with an ISS score between 16 and 24 with medical treatment	Run-specific, see Table 4		Kuwabara et al. (2010), Tyson et al. (2015)
prob_death_iss_25_35	The probability of mortality associated with an ISS score between 25 and 35 with medical treatment	Run-specific, see Table 4		Kuwabara et al. (2010), Tyson et al. (2015)
prob_death_iss_35_plus	The probability of mortality associated with an ISS score greater than 35 with medical treatment	Run-specific, see Table 4		Kuwabara et al. (2010), Tyson et al. (2015)
prob_death_MAIS1	The probability of death associated with a MAIS score of 1	0		Champion et al. (2010)
prob_death_MAIS2	The probability of death associated with a MAIS score of 2	0		Champion et al. (2010)
prob_death_MAIS3	The probability of death associated with a MAIS score of 3	0.05		Champion et al. (2010)
prob_death_MAIS4	The probability of death associated with a MAIS score of 4	0.31		Champion et al. (2010)
prob_death_MAIS5	The probability of death associated with a MAIS score of 5	0.59		Champion et al. (2010)
prob_death_MAIS6	The probability of death associated with a MAIS score of 6	0.83		Champion et al. (2010)
prob_perm_disability_with_treatment_severe_TBI	The probability that a person with a traumatic brain injury will be left permanently disabled	0.199		Eaton et al. (2017)
prob_perm_disability_with_treatment_sci	The probability that a person with a traumatic brain injury will be left permanently disabled	0.436		Eaton et al. (2019)

#### Number of injuries assigned per person

We fit the distribution to the following (*x*, *y*) data points: (1, % single injuries), (2, (% multiple injuries)/2) and (9, 0). We ran the model with several of the resulting distributions using Azure batch computing and calculated the average number of injuries in RTI hospital patients; the resulting number of distributions produced a 71:29 to 76:24 single-to-multiple injury ratio depending on other model parameter values (Table 3).

**Table 3:** A list of the injuries modelled in the road traffic injuries model, with the AIS score associated with the injury and DALY weight used to represent the health burden of that injury, the treatment plans designed to treat the injury and the source of information used to determine whether this treatment should be included

Injury	AIS score	Disability weight	Treatment	Recovery time	Source for use of treatment
‘Unspecified’ skull fracture	2	0.195	Heal with time or major surgery for depressed skull fracture	42 days with surgery, 49 otherwise	Eaton et al. (2017)
Basilar skull fracture	3	0.149	Heal with time	49 days	Assumed
Subarachnoid hematoma	3	0.214	Heal with time or major surgery (craniotomy)	42 days with surgery otherwise permanent	Eaton et al. (2017), Lavy et al. (2007)
Brain contusion	3	0.174	Heal with time or major surgery (craniotomy)	42 days with surgery otherwise permanent	Eaton et al. (2017), Lavy et al. (2007)
Intraventricular haemorrhage	3	0.174	Heal with time or major surgery (craniotomy)	42 days with surgery otherwise permanent	Eaton et al. (2017), Lavy et al. (2007)
Subgaleal hematoma	3	0.174	Heal with time or major surgery (craniotomy)	42 days with surgery otherwise permanent	Eaton et al. (2017), Lavy et al. (2007)
Epidural hematoma	4	0.185	Heal with time or major surgery (craniotomy)	42 days with surgery otherwise permanent	Eaton et al. (2017), Lavy et al. (2007)
Subdural hematoma	4	0.21	Heal with time or major surgery (craniotomy)	42 days with surgery otherwise permanent	Eaton et al. (2017), Lavy et al. (2007)
Diffuse axonal injury/midline shift	5	0.239	Heal with time or major surgery (craniotomy)	42 days with surgery otherwise permanent	Eaton et al. (2017), Lavy et al. (2007)
Laceration to the head	1	0.1	Suture kit, tetanus vaccine	7 days	Malawian treatment guidelines
Burn to the head	4	0.455	Cetrimide 15% + chlorhexidine 1.5% solution, Paraffin dressing, ringer's lactate (Hartmann's solution), tetanus vaccine	28 days with treatment otherwise permanent	Malawian treatment guidelines
Facial fracture (nasal/unspecified)	1	0.067	Minor surgery	49 days	Mkandawire et al. (2008)
Facial fracture (mandible/zygomatic)	2	0.067	Minor surgery	49 days	Mkandawire et al. (2008)
Soft tissue injury to face	1	0.1	Minor surgery	7 days	Mkandawire et al. (2008)
Laceration to the face	1	0.1	Suture kit, tetanus vaccine	7 days	Malawian treatment guidelines
Burn to the face	4	0.455	Cetrimide 15% + chlorhexidine 1.5% solution, Paraffin dressing, ringer's lactate (Hartmann's solution), tetanus vaccine	28 days with treatment otherwise permanent	Malawian treatment guidelines
Eye injury	1	0.054	Minor surgery	7 days	Mkandawire et al. (2008)
Soft tissue injury in neck (vertebral artery laceration)	2	0.1	Major surgery	42 days	Lavy et al. (2007)
Soft tissue injury in neck (pharynx contusion)	3	0.1	Major surgery	42 days	Lavy et al. (2007)
Sternomastoid m. haemorrhage/Haemorrhage, supraclavicular triangle/Haemorrhage, posterior triangle/Anterior vertebral vessel haemorrhage/Neck muscle haemorrhage	1	0.1	Major surgery	7 days	Lavy et al. (2007)
Hematoma in carotid sheath/Carotid sheath haemorrhage	3	0.1	Major surgery	14 days	Lavy et al. (2007)
Atlanto-occipital subluxation	2	0.187	Heal with time or minor surgery	42 days	Mkandawire et al. (2008)
Atlanto-axial subluxation	3	0.187	Heal with time or minor surgery	42 days	Mkandawire et al. (2008)
Laceration to the neck	1	0.1	Suture kit, tetanus vaccine	7 days	Malawian treatment guidelines
Burn to the neck	3	0.135	Cetrimide 15% + chlorhexidine 1.5% solution, Paraffin dressing, ringer's lactate (Hartmann's solution), tetanus vaccine	28 days with treatment otherwise permanent	Malawian treatment guidelines
Fractured rib(s)	2	0.187	Heal with time	35 days	Assumed
Flail chest	4	0.211	Major surgery	365 days	Bach (2004)
Chest wall bruises/haematoma	1	0.143	Heal with time	14 days	Assumed
Haemothorax	3	0.143	Thoracoscopy	7 days if treated surgically, otherwise 14 days	Paediatric handbook for Malawi
Lung contusion	3	0.205	Thoracoscopy	42 days with surgery, otherwise 84 days	Paediatric handbook for Malawi
Diaphragm rupture	3	0.205	Thoracoscopy	42 days with surgery, otherwise 84 days	Paediatric handbook for Malawi
Chest wall lacerations/avulsions	1	0.143	Thoracoscopy	14 days	Paediatric handbook for Malawi
Surgical emphysema	2	0.164	Heal with time	14 days	Assumed
Closed pneumothorax/open pneumothorax	3	0.164	Thoracoscopy	14 days	Paediatric handbook for Malawi
Laceration to the thorax	1	0.1	Suture kit, tetanus vaccine	7 days	Malawian treatment guidelines
Burn to the thorax	3	0.135	Cetrimide 15% + chlorhexidine 1.5% solution, Paraffin dressing, ringer's lactate (Hartmann's solution), tetanus vaccine	28 days with treatment otherwise permanent	Malawian treatment guidelines
Injury to stomach/intestines/colon	2	0.182	Laparotomy	90 days	Lavy et al. (2007), Katundu et al. (2018)
Injury to spleen/Urinary bladder/Liver/Urethra/Diaphragm	3	0.182	Laparotomy	90 days	Lavy et al. (2007), Katundu et al. (2018)
Injury to kidney	4	0.182	Laparotomy	90 days	Lavy et al. (2007), Katundu et al. (2018)
Laceration to the abdomen	1	0.1	Suture kit, tetanus vaccine	7 days	Malawian treatment guidelines
Burn to the abdomen	3	0.135	Cetrimide 15% + chlorhexidine 1.5% solution, Paraffin dressing, ringer's lactate (Hartmann's solution), tetanus vaccine	28 days with treatment otherwise permanent	Malawian treatment guidelines
Fracture vertebrae	2	0.111	Heal with time	63 days	Assumed
Spinal cord injury at neck level without treatment	3, 4, 5, 6	0.748	No treatment provided	Permanent	Eaton et al. (2019)
Spinal cord injury below neck level without treatment	3, 4, 5	0.623	No treatment provided	Permanent	Eaton et al. (2019)
Fracture to Clavicle, scapula, humerus	2	0.035	Fracture casting	49 days with treatment, 70 without	Thomas et al. (2021)
Fracture to Hand/wrist short term with or without treatment	2	0.01	Fracture casting	49 days with treatment, 70 without	Thomas et al. (2021)
Fracture to Hand/wrist long term without treatment	2	0.014	Fracture casting	49 days with treatment, 70 without	Thomas et al. (2021)
Fracture to Radius/ulna short term with or without treatment	2	0.028	Fracture casting	49 days with treatment, 70 without	Thomas et al. (2021)
Fracture to Radius/ulna long term without treatment	2	0.043	Fracture casting	49 days with treatment, 70 without	Thomas et al. (2021)
Shoulder dislocation	2	0.062	Minor surgery or given sling	84 days	Thomas et al. (2021)
Amputated finger	2	0.005	Major surgery	Permanent	Grudziak et al. (2019)
Unilateral arm amputation with treatment	2	0.039	Major surgery	Permanent	Grudziak et al. (2019)
Unilateral arm amputation without treatment	2	0.118	Major surgery	Permanent	Grudziak et al. (2019)
Thumb amputation	2	0.011	Major surgery	Permanent	Grudziak et al. (2019)
Bilateral arm amputation with treatment	3	0.123	Major surgery	Permanent	Grudziak et al. (2019)
Bilateral arm amputation without treatment	3	0.383	Major surgery	Permanent	Grudziak et al. (2019)
Laceration to the upper extremity	1	0.1	Suture kit, tetanus vaccine	7 days	Malawian treatment guidelines
Burn to the upper extremity	3	0.135	Cetrimide 15% + chlorhexidine 1.5% solution, Paraffin dressing, ringer's lactate (Hartmann's solution), tetanus vaccine	28 days with treatment otherwise permanent	Malawian treatment guidelines
Foot fracture short term with or without treatment	1	0.026	Heal with time, open reduction, external fixation, internal fixation	63 days with treatment	Chagomerana et al. (2017)
Foot fracture long term without treatment	1	0.026	Heal with time, open reduction, external fixation, internal fixation	Permanent	Chagomerana et al. (2017)
Open fracture of the foot	3	0.126	Ceftriaxone, Cetrimide 15% + chlorhexidine 1.5% solution, paraffin gauze, suture kit, Metronidazole	213 days with treatment, 240 days without	Schade et al. (2020)
Fracture to patella, tibia, fibula, ankle, short term without treatment	2	0.05	Skeletal traction, open reduction, external fixation, internal fixation, fracture casting	63 days with treatment, 70 days without	Chagomerana et al. (2017)
Fracture to patella, tibia, fibula, ankle, long term without treatment	2	0.055	Skeletal traction, open reduction, external fixation, internal fixation, fracture casting	Permanent	Chagomerana et al. (2017)
Open fracture of the patella, tibia, fibula, ankle	3	0.15	Ceftriaxone, Cetrimide 15% + chlorhexidine 1.5% solution, paraffin gauze, suture kit, Metronidazole	213 days	Schade et al. (2020)
Hip fracture short term with or without treatment	3	0.258	Major surgery	270 days	Lavy et al. (2007)
Hip fracture long term with treatment	3	0.058	Major surgery	Permanent	Lavy et al. (2007)
Hip fracture long term without treatment	3	0.402	Major surgery	Permanent	Lavy et al. (2007)
Pelvis fracture, short term	3	0.279	Major surgery	70 days	Lavy et al. (2007)
Pelvis fracture, long term	3	0.182	Major surgery	Permanent	Lavy et al. (2007)
Open fracture of the pelvis	3	0.379	Ceftriaxone, Cetrimide 15% + chlorhexidine 1.5% solution, paraffin gauze, suture kit, Metronidazole	213 days	Schade et al. (2020)
Femur fracture, short term with or without treatment	3	0.111	Skeletal traction, open reduction, external fixation, internal fixation, fracture casting	120 days	Chagomerana et al. (2017)
Femur fracture, long term without treatment	3	0.042	Skeletal traction, open reduction, external fixation, internal fixation, fracture casting	Permanent	Chagomerana et al. (2017)
Open fracture of the femur	3	0.211	Ceftriaxone, Cetrimide 15% + chlorhexidine 1.5% solution, paraffin gauze, suture kit, Metronidazole	213 days	Schade et al. (2020)
Dislocated hip	2	0.016	Skeletal traction, open reduction, external fixation, fracture casting	270 days	Chagomerana et al. (2017)
Dislocated Knee	2	0.113	fracture casting	49 days	Chagomerana et al. (2017)
Amputation of toes	2	0.006	Major surgery	Permanent	Grudziak et al. (2019)
Unilateral leg amputation with treatment	3	0.039	Major surgery	Permanent	Grudziak et al. (2019)
Unilateral leg amputation without treatment	3	0.173	Major surgery	Permanent	Grudziak et al. (2019)
Bilateral leg amputation with treatment	4	0.088	Major surgery	Permanent	Grudziak et al. (2019)
Bilateral leg amputation without treatment	4	0.443	Major surgery	Permanent	Grudziak et al. (2019)
Laceration to the lower extremity	1	0.1	Suture kit, tetanus vaccine	7 days	Malawian treatment guidelines
Burn to the lower extremity	3	0.135	Cetrimide 15% + chlorhexidine 1.5% solution, Paraffin dressing, ringer's lactate (Hartmann's solution), tetanus vaccine	28 days with treatment otherwise permanent	Malawian treatment guidelines

AIS scores were found using the R package ‘InjurySeverityScore’ which converted ICD-9 codes to their corresponding AIS scores, and DALY weights were taken from the GBD study and Gabbe et al. (2016). The sources for the treatment plans modelled are given within the table

An example of one probability distribution and resulting number of injuries in and out of hospital per person compared to the results of Sundet et al. (2018) is given in Fig. 7.

#### Determining the number of runs

To determine the number of runs we should use for each of the scenarios tested in the model, we aimed to find a small number of model runs which produced results which reliably fell within the ranges of our calibration targets. We began by running the model for a total of 20 model runs, each over the course of 10 years with a population of 20,000. We then sampled from these runs with an increasing sample size, selecting N runs at random for *N* = 1, 2, …, 20. We calculated the mean incidence of road traffic injuries between each run for each sample size and then repeated this process 100 times for each sample size. We decided on 4 runs per scenario as the mean incidence of road traffic injuries consistently fell within the 95% uncertainty interval reported in the GBD study (Table 4) (Fig. 8).

**Table 4:** As we had no specific calibration target for overall health seeking behaviour, other than a range of values from Zafar et al. (2018), we had to establish a parameter space for ‘rt_emergency_care_ISS_score_cut_off’, a parameter which influences the overall level of health seeking behaviour in the model

Emergency care ISS score cut-off	Base rate of injury, calibrated for each value of the emergency ISS cut-off score to produce an incidence of road traffic injuries equal to the average GBD estimates from 2010 to 2019	Number of injury distribution, calibrated for each value of the emergency ISS cut-off score to produce an average number of injuries for those who sought care as was reported in Sundet et al. (2018)		Probability of in-hospital mortality for the ISS score boundary per run	
1	0.0044	1	0.7094	ISS < 9	0.011
		2	0.2062	10 ≤ ISS ≤ 15	0.016
		3	0.0599	16 ≤ ISS ≤ 24	0.048
		4	0.0174	25 ≤ ISS ≤ 35	0.204
		5	0.0051	ISS > 35	0.346
		6	0.0015		
		7	0.0004		
		8	0.0001		
2	0.0043	1	0.7094	ISS < 9	0.009
		2	0.2062	10 ≤ ISS ≤ 15	0.013
		3	0.0599	16 ≤ ISS ≤ 24	0.037
		4	0.0174	25 ≤ ISS ≤ 35	0.156
		5	0.0051	ISS > 35	0.266
		6	0.0015		
		7	0.0004		
		8	0.0001		
3	0.0044	1	0.7094	ISS < 9	0.008
		2	0.2062	10 ≤ ISS ≤ 15	0.012
		3	0.0599	16 ≤ ISS ≤ 24	0.034
		4	0.0174	25 ≤ ISS ≤ 35	0.146
		5	0.0051	ISS > 35	0.248
		6	0.0015		
		7	0.0004		
		8	0.0001		
4	0.0043	1	0.7094	ISS < 9	0.008
		2	0.2062	10 ≤ ISS ≤ 15	0.013
		3	0.0599	16 ≤ ISS ≤ 24	0.036
		4	0.0174	25 ≤ ISS ≤ 35	0.154
		5	0.0051	ISS > 35	0.262
		6	0.0015		
		7	0.0004		
		8	0.0001		
5	0.0041	1	0.76127	ISS < 9	0.007
		2	0.18174	10 ≤ ISS ≤ 15	0.013
		3	0.04339	16 ≤ ISS ≤ 24	0.031
		4	0.01036	25 ≤ ISS ≤ 35	0.131
		5	0.00247	ISS > 35	0.223
		6	0.00059		
		7	0.00014		
		8	0.00003		
6	0.0041	1	0.76127	ISS < 9	0.006
		2	0.18174	10 ≤ ISS ≤ 15	0.009
		3	0.04339	16 ≤ ISS ≤ 24	0.027
		4	0.01036	25 ≤ ISS ≤ 35	0.114
		5	0.00247	ISS > 35	0.194
		6	0.00059		
		7	0.00014		
		8	0.00003		

For each value of ‘rt_emergency_care_ISS_score_cut_off’, we had to calibrate specific parts of the model to match our model’s outputs to the other calibration targets, such as the overall incidence of road traffic injuries, the average number of injuries per person in hospital and the overall level of in-hospital mortality. This table shows the parameter value used for ‘base_rate_injrti’, ‘number_of_injured_body_regions_distribution’, ‘prob_death_iss_less_than_9’, ‘prob_death_iss_10_15’, ‘prob_death_iss_16_24’, ‘prob_death_iss_25_35’ and ‘prob_death_iss_35_plus’ for each value of ‘rt_emergency_care_ISS_score_cut_off’ in our simulations

## Abbreviations

AIS: Abbreviated injury severity score
A&E: Accident and emergency
CHAI: Clinton Health Access Initiative
DALYs: Disability-adjusted life years
EHP: Essential health program
GBD: Global Burden of Disease Study
HSB: Health seeking behaviour
ISS: Injury severity score
IHS: Integrated household surveys
IBM: Individual-based model
KCH: Kamuzu Central Hospital
LMIC: Low- and middle-income countries
MAIS: Military abbreviated injury severity score
RTI: Road traffic injuries
SSA: Sub-Saharan Africa
TLO: Thanzi La Onse
WHO: World Health Organization

## References

[CR1] Abbafati C, Abbas KM, Abbasi-Kangevari M, Abd-Allah F, Abdelalim A, Abdollahi M, Abdollahpour I (2020). Global burden of 369 diseases and injuries in 204 countries and territories, 1990–2019: a systematic analysis for the global burden of disease study 2019. Lancet.

[CR2] Akinpelu OV, Oladele OA, Amusa YB, Ogundipe OK, Adeolu AA, Komolafe EO (2007). Review of road traffic accident admissions in a Nigerian Tertiary Hospital. Revi Road Traffic Acc Admin Nigeri Tertiary Hosp.

[CR3] Bach O (2004). Musculo skeletal trauma in an east African Public Hospital. Injury.

[CR4] Baker SP, O’Neill B, Haddon W, Long WB (1974). The injury severity score: a method for describing patients with multiple injuries and evaluating emergency care. J Trauma.

[CR5] Banza LN, Gallaher J, Dybvik E, Charles A, Hallan G, Gjertsen J-E, Mkandawire N, Varela C, Young S (2018). The rise in road traffic injuries in Lilongwe, Malawi a snapshot of the growing epidemic of trauma in low income countries. Int J Surg Open.

[CR6] Bhalla K, Harrison J, Shahraz S, Abraham J, Bartels D, Yeh PH, Naghavi M (2013). Burden of injuries in sub-Saharan Africa.

[CR7] Biluts H, Mersha A, Tsegazeab L, Abenezer T, Addisalem B (2015). Pattern of spine and spinal cord injuries in Tikur Anbessa Hospital, Ethiopia. Ethiopian Med J.

[CR8] Bun E (2012). Road traffic accidents in Nigeria: a public health problem. Afrimedic J.

[CR9] Carroll CP, Cochran JA, Price JP, Guse CE, Wang MC. The AIS-2005 revision in severe traumatic brain injury: Mission accomplished or problems for future research?” In Annals of advances in automotive medicine - 54th annual scientific conference;2010. p. 233–38. PMC3242550 21050606

[CR10] Chagomerana MB, Tomlinson J, Young S, Hosseinipour MC, Banza L, Lee CN (2017). High morbidity and mortality after lower extremity injuries in Malawi: a prospective cohort study of 905 patients. Int J Surg.

[CR11] Champion HR, Holcomb JB, Lawnick MM, Kelliher T, Spott MA, Galarneau MR, Jenkins DH (2010). Improved characterization of combat injury. J Trauma Inj Infect Crit Care.

[CR12] Chokotho L, Croke K, Mohammed M, Mulwafu W, Bertfelt J, Karpe S, Milusheva S (2022). Epidemiology of adult trauma injuries in Malawi: results from a multisite trauma registry. Injury Epidemiol.

[CR13] Court-Brown CM, Bugler KE, Clement ND, Duckworth AD, McQueen MM (2012). The epidemiology of open fractures in adults. A 15-year review. Injury.

[CR14] Eaton J, Hanif AB, Grudziak J, Charles A (2017). Epidemiology, management, and functional outcomes of traumatic brain injury in Sub-Saharan Africa. World Neurosurg.

[CR15] Eaton J, Mukuzunga C, Grudziak J, Charles A (2019). Characteristics and outcomes of traumatic spinal cord injury in a low-resource setting. Trop Doct.

[CR16] Gabbe BJ, Lyons RA, Harrison JE, Rivara FP, Ameratunga S, Jolley D, Polinder S, Derrett S (2014). Validating and improving injury burden estimates study: the injury-VIBES study protocol. Injury Prevent.

[CR17] Gabbe BJ, Lyons RA, Simpson PM, Rivara FP, Ameratunga S, Polinder S, Derrett S, Harrison JE (2016). Disability weights based on patient-reported data from a multinational injury cohort. Bull World Health Organ.

[CR18] Ganveer GB, Tiwari RR (2005). Injury pattern among non-fatal road traffic accident cases: a cross-sectional study in Central India. Indian J Med Sci.

[CR19] Gennarelli TA, Wodzin E (2006). AIS 2005: a contemporary injury scale. Injury.

[CR20] Global Health Data. GBD results tool | GHDx. Institue for Health Metrics and Evaluation; 2017.

[CR21] Government of Malawi. National Roads Authority Act. Malawi; 1997.

[CR22] Government of Malawi. Malawi human resources for health strategic plan; 2018. p. 2018–2022.

[CR23] Grudziak J, Mukuzunga C, Melhado C, Young S, Banza L, Cairns B, Charles A (2019). Etiology of major limb amputations at a tertiary care centre in Malawi. Malawi Med J.

[CR24] Haagsma JA, Graetz N, Bolliger I, Naghavi M, Higashi H, Mullany EC, Abera SF (2016). The global burden of injury: incidence, mortality, disability-adjusted life years and time trends from the global burden of disease study 2013. Inj Prev.

[CR25] Hassan S. Major injury cases in Nairobi: characterization of contexts, outcomes and injury documentation. University of Nairobi; 2016.

[CR26] Kasantikul V, Ouellet JV, Smith TA (2003). Head and neck injuries in fatal motorcycle collisions as determined by detailed autopsy. Traffic Inj Prev.

[CR27] Katundu KGH, Mutafya TW, Lozani NC, Nyirongo PM, Uebele ME (2018). An observational study of perioperative nutrition and postoperative outcomes in patients undergoing laparotomy at Queen Elizabeth Central Hospital in Blantyre, Malawi. Malawi Med J.

[CR28] Kuwabara K, Matsuda S, Imanaka Y, Fushimi K, Hashimoto H, Ishikawa KB, Horiguchi H (2010). Injury severity score, resource use, and outcome for trauma patients within a Japanese Administrative Database. J Trauma Inj Infect Crit Care.

[CR29] Lagarde E (2007). Road traffic injury is an escalating burden in Africa and deserves proportionate research efforts. PLoS Med.

[CR30] Lavy C, Tindall A, Steinlechner C, Mkandawire N, Chimangeni S (2007). Surgery in Malawi—a national survey of activity in rural and urban hospitals. Ann R Coll Surg Engl.

[CR31] Lee JS, Kim YH, Yun JS, Jung SE, Chae CS, Chung MJ (2016). Characteristics of patients injured in road traffic accidents according to the new injury severity score. Ann Rehabil Med.

[CR32] Lozano R, Naghavi M, Foreman K, Lim S, Shibuya K, Aboyans V, Abraham J (2012). Global and regional mortality from 235 causes of death for 20 age groups in 1990 and 2010: a systematic analysis for the global burden of disease study 2010. Lancet.

[CR33] Madubueze CC, Chukwu COO, Omoke NI, Oyakhilome OP, Ozo C (2011). Road traffic injuries as seen in a Nigerian Teaching Hospital. Int Orthop.

[CR34] Malm S, Maria K, Anders K, Anders Y, Claes T. Risk of permanent medical impairment (RPMI) in road traffic accidents. In Annals of advances in automotive medicine—52nd annual scientific conference, vol 52; 2008. p. 93–100. PMC3256772 19026226

[CR35] Mathers CD, Loncar D (2006). Projections of global mortality and burden of disease from 2002 to 2030. Edited by Jon Samet. Plos Med.

[CR36] Ministry of Health. Malawi standard treatment guidelines; 2015.

[CR37] Mkandawire N, Ngulube C, Lavy C (2008). Orthopaedic clinical officer program in Malawi: a model for providing orthopaedic care. Clin Orthop Relat Res.

[CR38] Mulima G, Purcell LN, Maine R, Bjornstad EC, Charles A (2021). Epidemiology of prehospital trauma deaths in Malawi: a retrospective cohort study. Afr J Emerg Med.

[CR39] Nasrullah M, Muazzam S (2012). Risk differences between children and adults in road traffic injuries: a descriptive study from a tertiary-care hospital in a low-income country. Eur J Emerg Med.

[CR40] National Statistical Office. Fifth integrated household survey 2019–2020 Malawi; 2021. https://microdata.worldbank.org/index.php/catalog/3818.

[CR41] Ngambi W, Tara M, Andrew P, Tim C, Joseph MB, Paul R, Hallett TB (2020). Factors associated with healthcare seeking behaviour for children in Malawi: 2016. Trop Med Int Health.

[CR42] Ngambi W, Tara M, Andrew PS, Tim C, Dominic N, Joseph M, Revill P, Hallett TB (2020). A cross-sectional study on factors associated with health seeking behaviour of malawians aged 15+ years in 2016. Malawi Med J.

[CR43] Ngwira GM, Benjamin B, Bhagabat PP (2020). Investigating the availability and usage of seatbelts in malawi for policy review and formulation. J Road Saf.

[CR44] Okugbo S, Okoro E, Irhibogbe P (2012). Chest trauma in a regional trauma centre. J West Afr College Surg.

[CR45] Otieno T, Woodfield JC, Bird P, Hill AG (2004). Trauma in rural Kenya. Injury.

[CR46] Ranti BO, Saheed Y, Adebayo OO, Kehinde O (2015). Injury pattern among patients with road traffic crash presenting at a tertiary health facility. IOSR J Dent Med Sci IOSR-JDMS.

[CR47] Ruhinda G, Kyamanywa P, Kitya D, Bajunirwe F (2008). Abdominal injury at Mbarara Regional Referral Hospital, Uganda. East Cent Afr J Surg.

[CR48] Salomon JA, Haagsma JA, Davis A, Maertens C, de Noordhout S, Polinder AH, Havelaar AC (2015). Disability weights for the global burden of disease 2013 study. Lancet Glob Health.

[CR49] Samuel JC, Sankhulani E, Qureshi JS, Baloyi P, Thupi C, Lee CN, Miller WC, Cairns BA, Charles AG (2012). Under-reporting of road traffic mortality in developing countries: application of a capture-recapture statistical model to refine mortality estimates. Edited by Vittoria Colizza. PLoS ONE.

[CR50] Sanyang E, Peek-Asa C, Bass P, Young TL, Daffeh B, Fuortes LJ (2017). Risk factors for road traffic injuries among different road users in the Gambia. J Environ Public Health.

[CR51] Sawe HR, Milusheva S, Croke K, Karpe S, Mohammed M, Mfinanga JA (2021). Burden of road traffic injuries in Tanzania: one-year prospective study of consecutive patients in 13 multilevel health facilities”. Edited by Piergiorgio Fedeli. Emerg Med Int.

[CR52] Schade AT, Yesaya M, Bates J, Martin C, Harrison WJ (2020). The Malawi Orthopaedic Association/Ao alliance guidelines and standards for open fracture management in Malawi: a national consensus statement. Malawi Med J.

[CR53] Schlottmann F, Tyson AF, Cairns BA, Varela C, Charles AG (2017). Road traffic collisions in Malawi: trends and patterns of mortality on scene. Malawi Med J.

[CR54] Singogo E, Kanike E, van Lettow M, Cataldo F, Zachariah R, Bissell K, Harries AD (2013). Village registers for vital registration in rural Malawi. Tropical Med Int Health.

[CR55] Staton CA, Joao R, Nickenig V, Nicole T, Jihad A, Patricia C, Michael H, Blandina TM, Mark M, Monica S (2018). The impact of alcohol among injury patients in Moshi, Tanzania: a nested case-crossover study. BMC Public Health.

[CR56] Stephan K, Huber S, Häberle S, Kanz KG, Bühren V, Van Griensven M, Meyer B, Biberthaler P, Lefering R, Huber-Wagner S (2015). Spinal cord injury—incidence, prognosis, and outcome: an analysis of the traumaregister DGU. Spine Journal.

[CR57] Sundet M, Grudziak J, Charles A, Banza L, Varela C, Young S (2018). Paediatric road traffic injuries in Lilongwe, Malawi: an analysis of 4776 consecutive cases. Trop Doct.

[CR58] Sundet M, Kajombo C, Mulima G, Bogstrand ST, Varela C, Young S, Christophersen AS, Gjerde H (2020). Prevalence of alcohol use among road traffic crash victims presenting to a malawian central hospital: a cross-sectional study. Traffic Inj Prev.

[CR59] Thanni LOA, Kehinde OA (2006). Trauma at a Nigerian teaching hospital: pattern and documentation of presentation. Afr Health Sci.

[CR60] Thomas A, Schade I, Foster M, Paul C, Peter MI, Simon MG, Claude M, William JH, Linda C. Epidemiology of fractures and their treatment in Malawi: results of a multicentre prospective registry study to guide orthopaedic care planning; 2021. 10.1371/journal.pone.0255052. 10.1371/journal.pone.0255052 PMC8336825 34347803

[CR61] Tian D. Injury severity score; 2019.

[CR62] Tian H, Wang L, Xie W, Shen C, Guo G, Liu J, Han C (2018). Epidemiologic and clinical characteristics of severe burn patients: results of a retrospective multicenter study in China, 2011–2015. Burns Trauma.

[CR63] Tyson AF, Varela C, Cairns BA, Charles AG (2015). Hospital mortality following trauma: an analysis of a hospital-based injury surveillance registry in Sub-Saharan Africa. J Surg Educ.

[CR64] Vos T, Barber RM, Bell B, Bertozzi-Villa A, Biryukov S, Bolliger I, Charlson F (2015). Global, regional, and national incidence, prevalence, and years lived with disability for 301 acute and chronic diseases and injuries in 188 countries, 1990–2013: a systematic analysis for the global burden of disease study 2013. Lancet.

[CR65] WHO (2005). Malawi world health survey 2003.

[CR66] WHO (2015). Global status report on road safety 2015.

[CR67] WHO (2018). Global status report on road safety 2018.

[CR68] Zafar SN, Canner JK, Nagarajan N, Kushner AL, Shailvi Gupta TuM, Tran NN (2018). Road traffic injuries: cross-sectional cluster randomized countrywide population data from 4 low-income countries. Int J Surg.

